# Proteomic characterization of primary cultured myocytes in a fish model at different myogenesis stages

**DOI:** 10.1038/s41598-019-50651-w

**Published:** 2019-10-01

**Authors:** Antonio F. Millan-Cubillo, Miguel Martin-Perez, Antoni Ibarz, Jaume Fernandez-Borras, Joaquim Gutiérrez, Josefina Blasco

**Affiliations:** 1 Departament of Cell Biology, Physiology and Immunology, Faculty of Biology, University of Barcelona, 08028 Barcelona, Spain; 2 Present Address: Institute for Research in Biomedicine (IRB Barcelona), The Barcelona Institute of Science and Technology, 08028 Barcelona, Spain

**Keywords:** Cell proliferation, Differentiation

## Abstract

Myogenesis is a complex two-phase process of proliferation and differentiation, which seems to be greatly conserved in vertebrates. For the first time in fish, we identify the changes that occur in the proteome during this process in a gilthead sea bream (*Sparus aurata*) myocyte primary cell culture (on days 4, 8 and 12), using 2-D gel electrophoresis and LC-MS/MS. A significant increase of myogenin expression at day 8 marked the transition from proliferation to differentiation. Of the 898 spots in the proteome analysis, the 25 protein spots overexpressed on day 4 and the 15 protein spots overexpressed on day 8 indicate the end of proliferation and the beginning of differentiation, respectively. Proliferation was characterized by enrichment of proteins involved in actin cytoskeleton remodelling and in cellular metabolic processes (transcription, ubiquitination, response to stress and glucose metabolism). During differentiation, 41 proteins were overexpressed and 51 underexpressed; many of them related to biosynthetic processes (RNA and protein synthesis and folding, and pentose pathways), terminal myotube formation and muscle contraction. The main cellular processes of both phases of muscle development in fish are similar with those observed in mammals but extended in time, allowing sequential studies of myogenesis.

## Introduction

Fish occupy a unique position among vertebrates when considering growth patterns and strategies. The development of muscle in fish differs from that of mammals and birds, because the formation of the different types of muscle fibres is spatially and temporally separated, and also because the formation of new muscle fibres continues well into the adult life of all fish species studied. The final wave of myogenesis in muscle begins in larval stage and continues until the fish reaches around 40% of its adult body length^1,2^. Both hypertrophic and hyperplastic muscle growth can take place concomitantly in fish throughout their lifecycle^1,3^, being modulated by several environmental and endogenous factors such as temperature, photoperiod, diet, or fish strain^4–9^. To avoid variation in external factors that affect development and growth, *in vitro* models provide a more stable framework^10^. The performance of the developing myotome, and especially its hyperplastic growth, involves the proper integration of three main cell processes: proliferation, differentiation, and death. Myogenesis results from the first two of these processes, proliferation and differentiation: the specification of some somitic cells as myosatellite cells, and their subsequent differentiation and fusion into mature myotubes^11,12^. These events are controlled by specific endocrine factors present in the blood and by several myogenic regulatory factors (MRFs) produced locally by the muscle cells themselves or by their neighbouring tissues^13^. Most of the molecular mechanisms involved in myogenesis are conserved throughout vertebrates and invertebrates^14,15^. Cell cycle withdrawal and the sequential expression of myogenic regulatory factors (MRFs) and muscle-specific genes are necessary for the differentiation process. So, a prerequisite for initiation of myoblast differentiation is growth arrest, which is stimulated by myostatin^16^. In mammals^17^, MyoD and Myf5 are involved in myogenic lineage determination, and Myogenin and MRF4 play key roles in initiating and maintaining myogenic differentiation, as has also been found in fish^10,13^. Although not as broadly studied as in mammals, there is now extensive knowledge of the muscle growth process in teleosts^18^ and we are beginning to understand the mechanisms regulating the developmental process through studies of regulatory genes with primary cultures of myocytes in gilthead sea bream^19^ or in trout^20,21^. Thus, in gilthead sea bream myogenic cultures, the *myod2* and *myf5* genes are up-regulated during the proliferation phase, while *myogenin* and *mrf4* are expressed more during the differentiation phase^19^, in agreement with what has been observed in mammals. However, it is necessary to study proteome performance through all the processes or stages in order to understand the molecular mechanisms underlying the phenotypic transition during myogenic differentiation, beyond regulatory factors. Proteomic tools have helped to determine the groups of proteins that are expressed in the development of myotubes in mammals, both in primary cultures of muscle cells^22^ in established cell lines^23–26^ and by complementing the profile of genes expressed during the process of myogenesis. This proteome work has provided a holistic framework to understand how different biochemical processes are coordinated at the cellular level: cell cycle exit, cell adhesion and migration, metabolism, proteolysis, extra cellular matrix (ECM) remodelling and fusion, and muscle contraction. To date, no studies have examined myocyte proteome expression during the main stages of myogenesis in fish. Although at the transcriptional level it is well known that the process of myogenesis is highly conserved, there is no information on the associated processes at the protein level.

The aim of the present work is to characterize the proteome of muscle culture cells of gilthead sea bream (*Sparus aurata*) in different myogenesis stages, from myoblast cells to myotubes at different times of primary culture: days 4, 8 and 12 and subsequently, to identify by 2-D gel electrophoresis and mass spectrometry the differentially expressed proteins. Our study recognises, for the first time in studies of fish primary cell cultures throughout myogenic processes, how distinct proteins grouped into protein clusters in relation to their main biological functions. Within the characteristic framework of fish myogenesis and from the proliferation to the differentiation phase, we observed abrupt changes in proteins related to the cell cycle activity, the establishment of cellular metabolism and of the muscle contractile properties, and muscle cell reorganization.

## Results and Discussion

### Characterization of cell culture development and proteome

**Figure 1:**
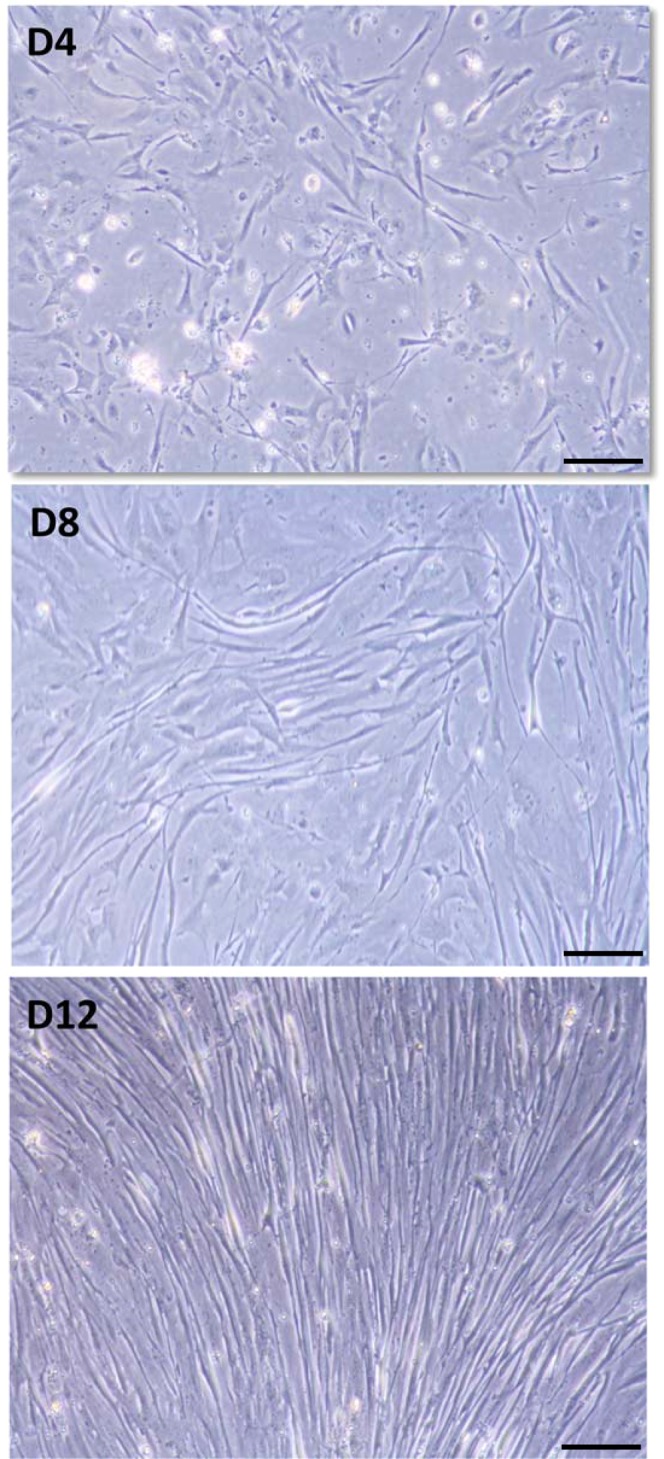
Differentiation of satellite cells isolated from skeletal muscle of gilthead sea bream and cultured on pre-treated plates with poly-L-lysine and laminin at 18 °C for 4 days (D4), 8 days (D8), and 12 days (D12). These images are of one representative sample of five independent culture. Scale bars: D4 to D12, 100 µm.

With the aim of characterizing muscle growth and development, in the present study we used a primary culture of gilthead sea bream myocytes as our study model. Representative images of cell phenotypes at different days of culture are shown in Fig. 1; these cell phenotypes are in agreement with our previous observations in primary cultures of muscle cells in this species^19,27^, in spite of the fact that there is a certain degree of variability between cultures when dealing with primary cultures. In these studies, we observed that between days 2 and 4 after seeding, cells are mononucleated; while between days 6 and 8, cells start to differentiate, becoming myotubes at day 12 and at day 15. So, in the present work, at day 4 (D4, Fig. 1A), when mononucleated satellite cells undergoing proliferation are uniformly distributed, most cells adopted an elongated shape. As development continues, the cells merged together to form small myotubes during the proliferative phase which lasts until day 8 (D8, Fig. 1B). Subsequently, the cells differentiated and become multinucleated myotubes with the highest confluence on day 12 (D12, Fig. 1C). In a previous work^27^, it quantified by DAPI staining that 80% of the cells in this type of culture are polynucleated at 12 days.

**Figure 2:**
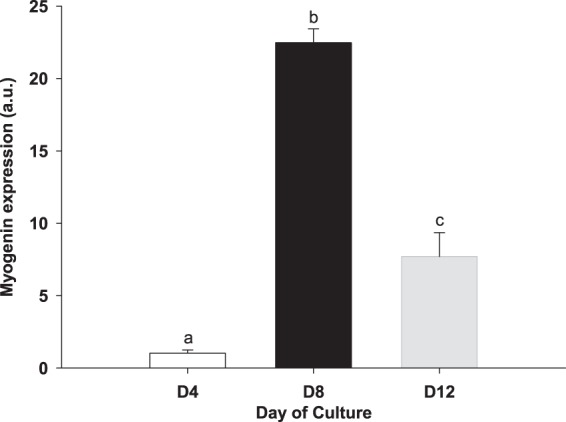
Expression of myogenin in cultured gilthead sea bream muscle cells. Myogenin expression was evaluated in myoblasts from day 1 to day 12 of culture by qPCR. Results are shown as mean ± SEM of 5 independent experiments. Values not sharing a common letter are significantly different (p < 0.05). (a.u.): arbitrary units.

Myogenin expression was used as a marker of the transition from the proliferation to the differentiation phase^19^. Myogenin expression increased twentyfold between D4 and D8, and decreased three times from D8 to D12 (Fig. 2), corroborating that the differentiation phase initiated at D8.

**Figure 3:**
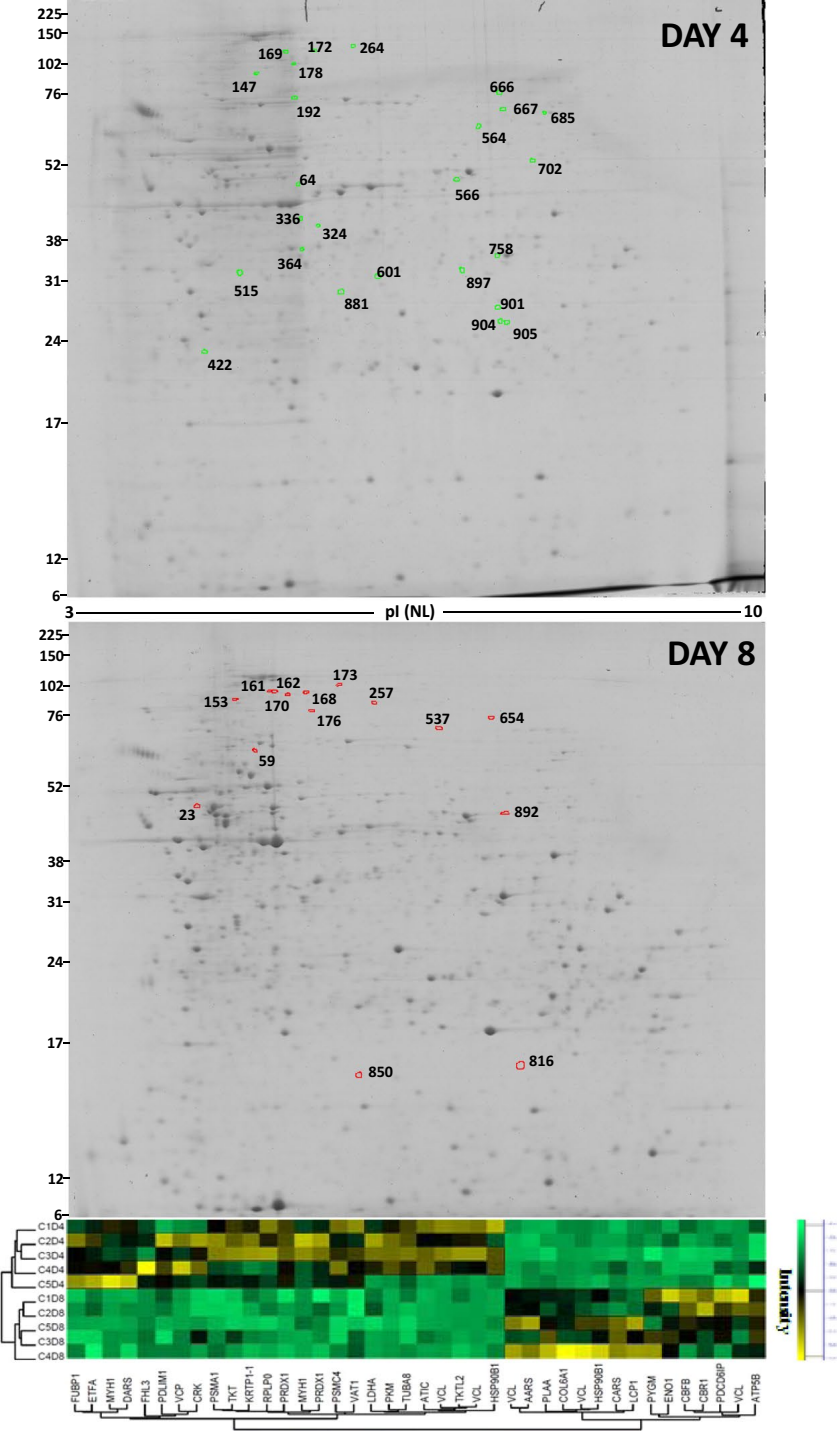
2-DE master gels map showing the protein expression profiles from day 4 and day 8. Gels are loaded with 300 μg of total protein extract from the soluble muscle cell protein fractions. Proteins were separated in the first dimension on pH 3–10 non-linear IPG strips, followed by SDS-PAGE on 12.5% w/v gels. Gels were stained with colloidal CBB. Candidate spots listed on the gels were identified by MS. The images of full-length gels are shown in Supplementary information (Fig. S1: C2D4 of day 4 and C5D8 of day 8). Spots outlined in red and green indicate the proteins that were overexpressed and underexpressed respectively. Below each gel, the expression data for selected spots are shown in agglomerative hierarchical clusters of Z-score transformed intensity values (Perseus program). Cluster coloration indicates protein abundance in the sample (yellow: higher abundance, green: lower abundance, black: unchanged). C1, C2, C3, C4 and C5 represent five culture and D4 and D8 represent the day of culture: 4 or 8 days.

**Figure 4:**
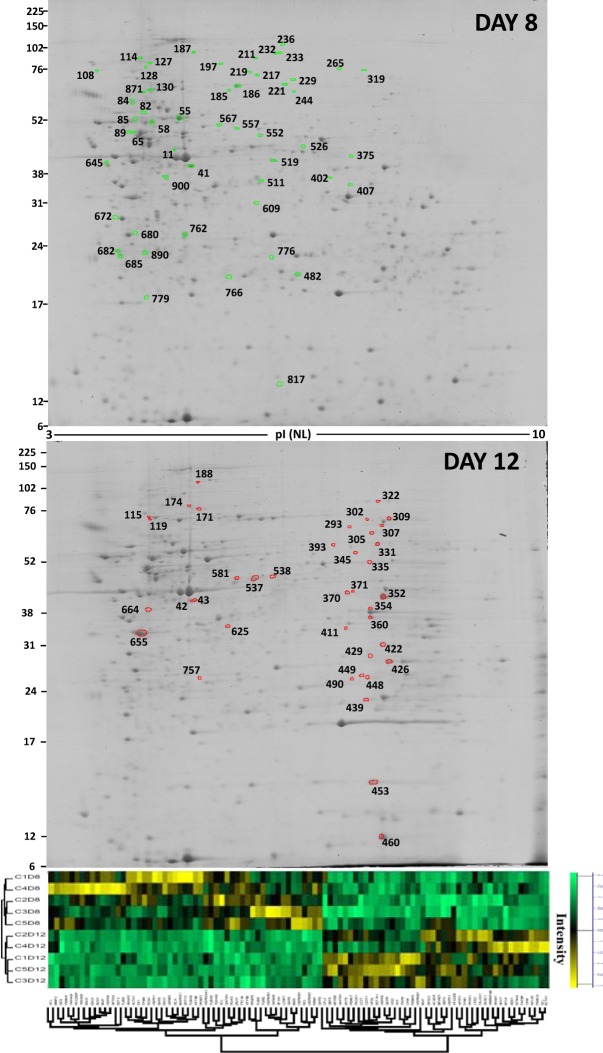
2-DE master gels map showing the protein expression profiles from day 8 and day 12. Details in Fig. 3. C1, C2, C3, C4 and C5 represent five culture and D8 and D12 represent the day of culture: 8 or 12 days. The images of full-length gels are shown in Supplementary information (Fig. S1: C5D8 of day 8 and C4D12 of day 12).

**Figure 5:**
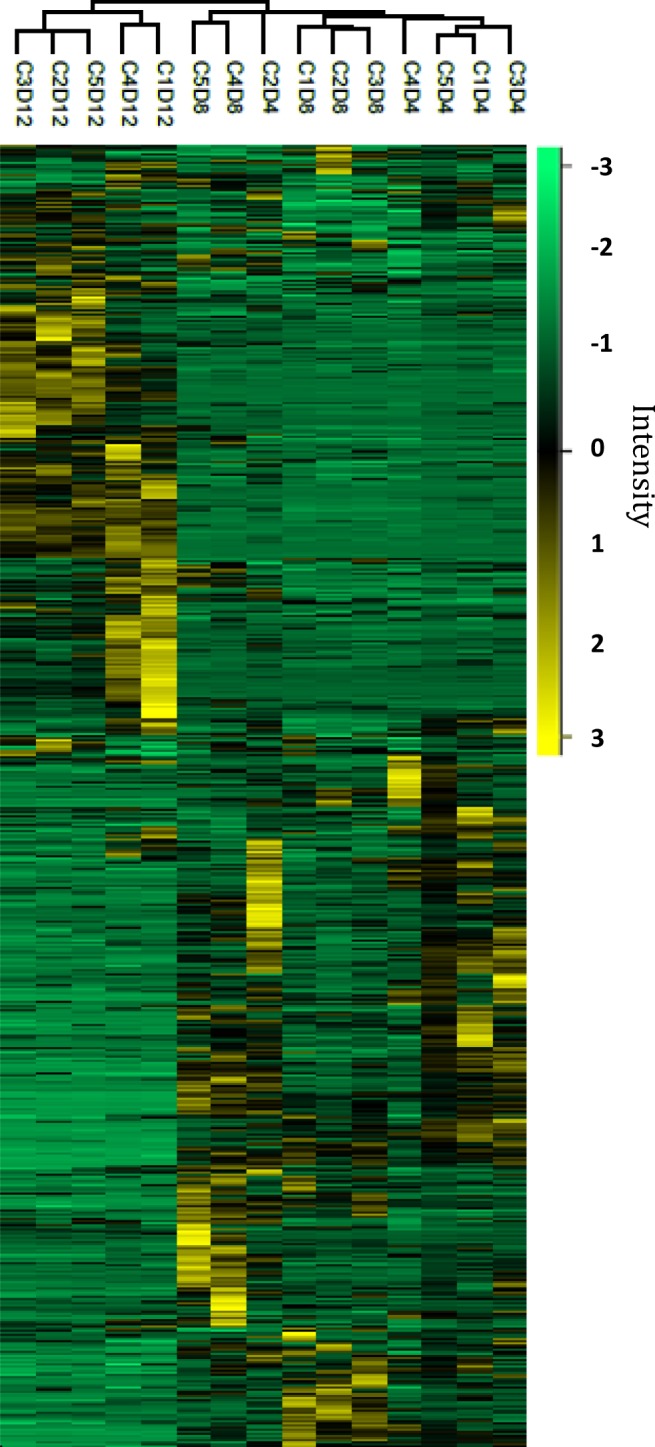
Heat map of the hierarchical clustering of the relative abundance of 898 matched spots on different days of muscular cell culturing. The vertical dendrogram represents the correlation distances between spot abundance levels (Perseus program). Every line represents one independent culture at one time-point, while the different spots represented by individual rows. Yellow indicates high levels of expression while green represents low levels. The intensity of the colours represents the relative abundance. C1, C2, C3, C4 and C5 represent five culture and D4, D8 and 12 days represent the day of culture: 4, 8 or 12 days. Raw data of each spot are included in Table S5 (Supplementary information). Individual gels are provided as Supplementary information (Gels C1D4 to C5D12).

Protein extracts from five independent biological replicates of primary cell cultures were obtained on D4, D8 and D12, to systematically characterize the sea bream muscle proteome using 2DE gel maps. D8 gels were used as master gels and compared against D4 gels (proliferation phase) (Fig. 3), and D12 gels (differentiation phase) (Fig. 4), with a total of 898 spots individually detected and matched among the 15 gels. The expression profiles of all the matched spots at each stage (D4, D8 and D12) were analysed by agglomerative hierarchical cluster (Fig. 5). Most samples within the same culture stage clustered together, except one replicate (C2D4) from D4 cultures that developed faster and was allocated within the D8 cultures.

One hundred thirty two proteins were differentially expressed, being distributed uniformly according to their mean relative abundance among the 898 spots (see Fig. S2 in supplementary information). During proliferation (D4 to D8), 40 spots (around 4% of the matched spots from the whole proteome) changed significantly: 15 protein spots were overexpressed and 25 were underexpressed at day 8 with respect to day 4 (Fig. 3, Supplementary Table S1). Over the differentiation phase (D8 *vs* D12) 92 spots were differentially expressed: 41were overexpressed and 51 underexpressed (Fig. 4, Supplementary Table S2). Identities of the spots analysed from the first and second developmental stages, along with information on each protein identified, and the fold change are all summarized in Supplementary Tables S1 and S2, respectively. All proteins identified had a teleost match.

**Figure 6:**
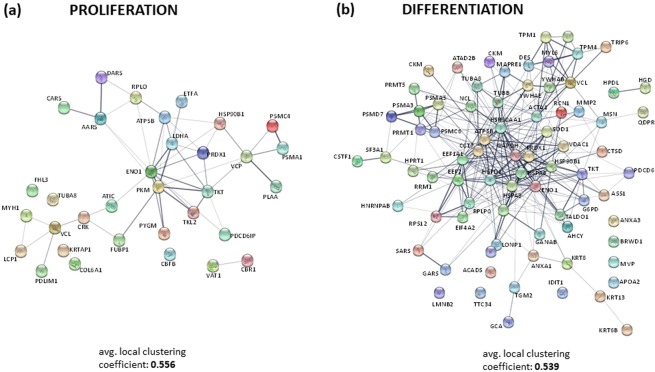
The protein–protein interaction network, the interactome, of proteins differentially expressed during the two developmental stages in a gilthead sea bream primary muscle cell culture: (**a**) Proliferation and (**b**) Differentiation. The average local clustering coefficients were 0.556 and 0.539 for each phase respectively, and more relevant data from the network stats are provided in supplementary information (Tables S3 and S4). In these networks, nodes are proteins, the thickness of the line indicate the degree of confidence prediction of the interaction, according to the STRING databases^66^. Protein acronyms correspond to Gene Symbol (for details, see Supplementary information: Table S1 (Proliferation) and Table S2 (Differentiation).

Functional protein-protein interaction networks of the differentially regulated proteins for each phase were generated from STRING analyses (Fig. 6: (a) proliferation and (b) differentiation). The resulting network of proteins in the proliferation and differentiation phases showed similar clustering coefficients (0.556 and 0.539, respectively), despite the differences in the number of differently expressed proteins through each phase. These coefficients are the measurement of how connected the nodes in the network are and the main functional clusters emerged within each network are detailed below.

### Protein expression in primary cultures during the proliferation phase

**Figure 7:**
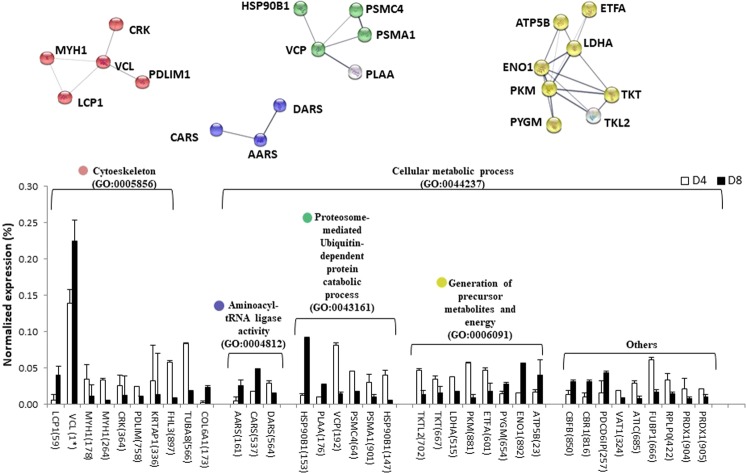
Functional clusters and abundance of differentially expressed proteins during the proliferation developmental stage (day 4 *vs* day 8) in a gilthead sea bream (*Sparus aurata* L.) primary muscle cell culture. On the top, the network analysis of protein-protein interactions among proteins grouped into 4 functional categories, according to Gene Ontology enrichment analysis by GOEAST. In this network, nodes are proteins, the thickness of the line indicate the degree of confidence prediction of the interaction, according to the STRING databases^66^. On the bottom, the relative abundance of protein spots of each cluster are showed. Data are shown as mean ± SEM of % vol. Significant differences by t-test (p < 0.05). The average of local clustering coefficients were: 0.521 for “Cytoeskeleton” (GO: 0005856, p = 0.00804), 0.667 for “Aminoacyl-tRNA ligase activity” (GO: 0004812, p = 1.02e-05), 0.833 for “proteosome-mediated ubiquitin-dependent protein catabolic process” (GO: 0043161, p = 0.000866), 0.805 for “Generation of precursor metabolites and energy” (GO: 0006091, p = 3.54e-07). Protein acronyms correspond to Gene Symbol (see Supplementary Table S1 for details).The interactome of proliferation phase is represented in Fig. 6A, and more relevant data from the network stats are provided in Supplementary information (Table S3).

The 40 differentially expressed proteins during proliferation were linked to a range of functional categories associated with cell structure or cell metabolism (Fig. 7 and Supplementary Table S1). The four clusters emerging from Gene Ontology enrichment analysis had different cluster coefficients, indicating differences in the interaction of proteins: 0.521 for Cytoskeleton (GO: 0005856); 0.667 for T-RNA aminoacylation for protein translation (GO: 0006418); 0.833 for Proteasome-mediated ubiquitin-dependent protein catabolic process (GO: 0043161) and 0.805 for Generation of precursor metabolites and energy (GO: 0006091) (see the relevant data from the network stats in Supplementary Table S3).

From D4 to D8, seven differentially expressed proteins corresponded to the “Cytoskeleton” cluster, being five of them highly interconnected (local cluster coefficient: 0.521): Among them we found two myosin heavy-chain forms (MYH1, spots 178 and 264), a LIM protein (FHL3) and α-tubulin. During the development of fish skeletal muscles, different myosin heavy chains are expressed in sequential fashion from early stages of larval development to adult muscle specific isoforms^28,29^. Thus, not only during ontogenesis, but also during *in vitro* myogenesis myosin heavy-chain isoforms change. FHL3 stimulates the myogenic progenitor cell population and skeletal muscle regeneration acting *fhi3* gen in a coordinated fashion with s*ox15* and *foxk1* in murine C2C12 cells^30^. In gilthead sea bream, overexpression of the *fhi2* gene has also been associated with the development of craniofacial musculature^31^, but the present work is the first report of the presence of LIM proteins directly related to cell culture development in fish. Alpha-tubulin, expressed in most organisms as the major constituent of microtubules, is involved in the intracellular processes of transport, cytokinesis, cell shape maintenance and migration^32^. During differentiation of human primary myoblast α-tubulin remained constant, as do many other housekeeping proteins^22^. So, the downregulation of α-tubulin should be a signal that the proliferation process is ending. Moreover, two proteins in the cytoskeleton cluster were upregulated at D8: plastin-2 (LCP, spot 59), an actin-binding protein; and vinculin (VCL, spots 162, 168, 169, 170, and 172), a protein involved in cell-matrix adhesion that plays roles in cell morphology and cadherin expression. The overexpression of these proteins indicates the beginning of cellular fusion. Skeletal muscle cells synthesize their own extracellular matrix during the transition from mononucleated myoblasts to syncytial myotubes^33,34^ and cell-matrix interactions facilitate myoblast fusion^35^. The upregulation of COL6A1^22^, a secretory form of collagen, and vinculin, localized in a perinuclear structure^36^ during the proliferation phase of human primary myoblasts, have also been observed. With its prominent links to the actin cytoskeleton, vinculin is a strong candidate as a mediator of the transmission of signals between the cell-matrix and the cytoskeleton^37^.

The D4 to D8 transition involves changes in proteins participating in protein synthesis (“aminoacyl-tRNA ligase activity”; GO: 0004812), and degradation (“ubiquitin dependent protein catabolic process”; GO: 0006511), indicating that the protein turnover is profoundly altered. Among them, several complex proteasome subunits (PSMC4 and PSCMA1, spots 64 and 901, respectively) were under expressed in D8. Valosin-containing protein (VCP, spot 192) was also downregulated. VCP is a multi-ubiquitin chain-targeting factor required in the degradation of many proteasome substrates^38^, confirming the decrease of protein turnover at day 8.

The 94-glucose-regulated protein (HSP90B1) is the most abundant chaperone in the endoplasmic reticulum^39^ showing an isoform modification from a more acid and putatively phosphorylated form (spot 147, observed pI = 4.4), which was downregulated at D8, to a more basic form (spot 153, observed pI = 4.7), upregulated at D8 (see Figs 3 and 7). As the active phosphorylated form is involved in cell proliferation^39,40^, the reduced amount of this protein in skeletal myoblasts should indicate the loss of fusion capacity^41^. So, in the present work, the decrease of the acidic isoform (likely phosphorylated) suggests the end of proliferation and the beginning of the differentiation phase.

The changes observed between the proteins grouped in the “generation of precursor metabolites and energy” cluster involved the downregulation between D4 and D8 of two transketolases (TKT, spot 667 and TKTL2, spot 702), alpha-pyruvate kinase (PKM, spot 881) and lactate dehydrogenase (LDH, spot 515), while glycogen phosphorylase (PYGM, spot 654) and enolase (ENO1, spot 892) were overexpressed. TKT, enzyme from the pentose pathway, provides the necessary amounts of nucleic acids for proliferation while downregulation of TKTL1 may be related with an inhibition of cell proliferation and the cell cycle arrests^42^. Meanwhile, the overexpression of proteins involved in glycolysis, such as enolase and glycogen phosphorylase, confirm the transition from proliferation to differentiation, characterized by an increase in the glycolytic machinery, as previously observed during early myogenesis in bovines^43^. Moreover, the overexpression of ATP synthase (ATP5B, spot 23) indicates a considerable increase in energy production via glucose catabolism at the beginning of the differentiation phase, in agreement with what is observed during C2C12 myoblast differentiation^25^.

A miscellaneous group of proteins were also modifying their expression in response to proliferation phase (Fig. 7). It is notable that peroxiredoxin 1 (spots 904 and 905, see others in Fig. 7), closely related with the regulation of cell proliferation^44^ was downregulated from D4 to D8. Phosphorylation of Prx1 occurs during mitosis, but not during the interphase^45^, and it is an important switch for the upregulation of cellular levels of hydrogen peroxide, resulting in the progression of the cell cycle. Another downregulated protein, ungrouped, was FUBP1 (spot 666) that participates in the regulation of *c-myc* in undifferentiated cells. Trumpp *et al*.^46^ defined *c-myc* as the senior administrator of cellular resources, controlling cell behaviour as diverse as proliferation, growth, differentiation, and apoptosis. Accurate levels of *c-myc* expression, and its timing, seem to be extremely important in mammals. There is no knowledge of FUBP1 in fish; but again, the downregulation of this factor at D8 would suggest this protein in a gradual stop of the proliferation phase via c-myc. Meanwhile, two proteins that had become upregulated at D8, the core binding factor beta (CBFB, spot 850) and the carbonyl reductase 1 (CBR1, spot 816), indicate that the proliferation phase was still not over and differentiation was only just starting. In this sense, the overexpression of CBFB in myoblasts of cell line C2D12 slows terminal differentiation via interaction with the master myogenic transcription factor MyoD^47^. A spontaneous decrease in the size of several tumours induced by CBR1 expression supports the idea that this protein acts as a development retardant^48^.

### Protein expression patterns in the differentiation phase

**Figure 8:**
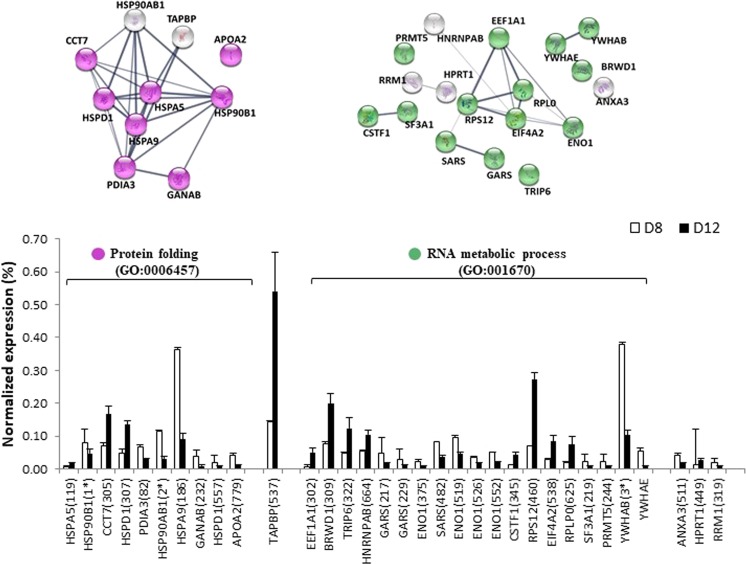
Relative abundance of differentially expressed proteins and functional clusters related with protein folding and RNA metabolic process during the differentiation developmental stage (day 8 *vs* day 12) in a gilthead sea bream primary muscle cell culture. On the top, the network analysis of protein-protein interactions among proteins grouped into two functional categories, according to Gene Ontology enrichment analysis by GOEAST. In these networks, nodes are proteins, the thickness of the line indicate the degree of confidence prediction of the interaction, according to the STRING^66^. On the bottom, the relative abundance of protein spots of each cluster are showed. Data are shown as mean ± SEM of % vol. Significant differences by t-test (p < 0.05). The average of local clustering coefficients were 0.813 for “Protein folding” (GO: 0006457, p = 4.08e-11) and 0.678 for “RNA metabolic process” (GO: 001670, p = 1.66e-05). Protein acronyms correspond to Gene Symbol (see Supplementary Table S2 for details).The interactome of differentiation phase is represented in Fig. 6B, and more relevant data from the network stats are provided in Supplementary information (Table S4).

**Figure 9:**
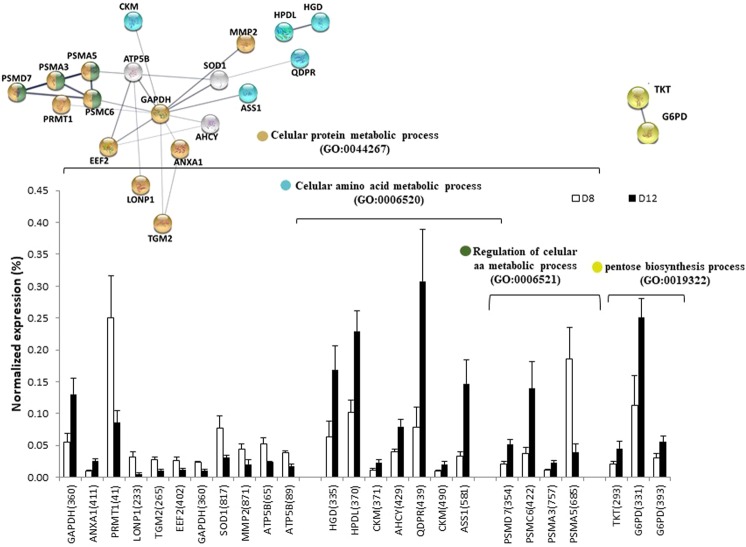
Relative abundance of differentially expressed proteins and functional clusters related with cellular protein metabolic process and pentose biosynthesis process during the differentiation developmental stage (day 8 vs day 12) in a gilthead sea bream primary muscle cell culture. On the top, the network analysis of protein-protein interactions among proteins grouped into four functional categories, according to Gene Ontology enrichment analysis by GOEAST. In these networks, nodes are proteins, the thickness of the line indicate the degree of confidence prediction of the interaction, according to the STRING databases^66^. On the bottom, the relative abundance of protein spots of each cluster are showed. Data are shown as mean ± SEM of % vol. Significant differences by t-test (p < 0.05). The average of local clustering coefficients were 0.79 for “Cellular protein metabolic process” (GO: 0044267, p = 4.95e-05), which grouped two clusters: “Cellular amino acid metabolic process” (GO: 0006520, p = 6.75e-05) and “Regulation of cellular aa metabolic process” (GO: 0006521, p = 1.41e-07, clustering coefficient = 1), and “pentose biosynthesis process” (GO: 0019322, p = 0.000395, clustering coefficient = 1). Protein acronyms correspond to Gene Symbol (see Supplementary Table S2 for details).The interactome of differentiation phase is represented in Fig. 6B, and more relevant data from the network stats are provided in Supplementary information (Table S4).

**Figure 10:**
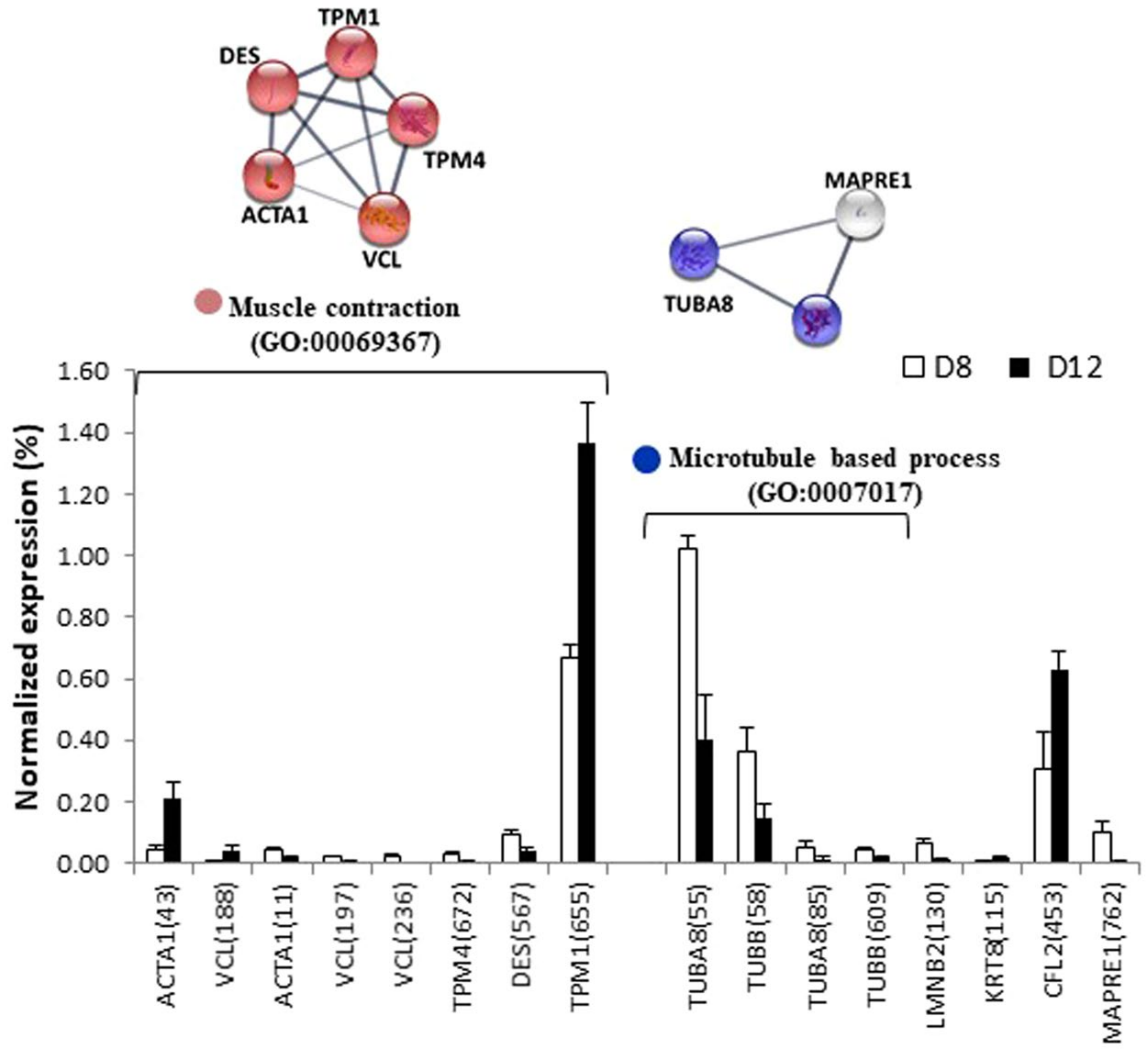
Relative abundance of differentially expressed proteins and functional clusters related with muscle contraction and microtubule based process during the differentiation developmental stage (day 8 vs day 12) in a gilthead sea bream primary muscle cell culture. On the top, the network analysis of protein-protein interactions among proteins grouped into two functional categories, according to Gene Ontology enrichment analysis by GOEAST. In these networks, nodes are proteins, the thickness of the line indicate the degree of confidence prediction of the interaction, according to the STRING databases^66^. On the bottom, the relative abundance of protein spots of each cluster are showed. Data are shown as mean ± SEM of % vol. Significant differences by t-test (p < 0.05). The average of local clustering coefficients were 1 for “Muscle contraction” (GO: 00069367, p = 3.85e-07) and for “Microtubule based process” (GO: 0007017). Protein acronyms correspond to Gene Symbol (see Supplementary Table S2 for details). The interactome of differentiation phase is represented in Fig. 6B, and more relevant data from the network stats are provided in Supplementary information (Table S4).

During differentiation, from D8 to D12, the differentially expressed protein spots distributed in eight functional clusters. Most of these proteins grouped in clusters (Supplementary Table S2) associated with protein synthesis and related processes (Figs 8 and 9), and only 7 proteins grouped in two clusters related to muscle contraction and cytoskeleton (Fig. 10). More relevant data from the network stats is shown in Supplementary Table S4.

In Fig. 8, two clusters are represented together with the percentage of expression of each of the proteins involved: “Protein folding” (GO: 0006457) and “RNA metabolic process” (GO: 001670), with interaction coefficients of 0.813 and 0.678, respectively. The first cluster grouped chaperones assist in the global process of recovery from stress, either by repairing damaged proteins (protein refolding) or by helping to degrade them and restore protein homeostasis, and so they promote cell survival. Among these proteins, two heat shock proteins were strongly underexpressed (HSP90B1, spot 114; HSP90AB1, spots 127and 128), but also overexpressed (HSP90B1, spot 174). There is little knowledge of the role played by each of the different isoforms during cellular development. In rainbow trout, the high expression levels of the gene *grp94a* indicated that this isoform (HSP90B1) is vitally required throughout the life cycle^49^. Nevertheless, the overexpression of some chaperone isoforms and underexpression of others indicate that the protein synthesis machinery is highly activated and that the proteolytic systems play important roles in remodelling intracellular components during myoblast differentiation^50,51^.

The process of eukaryotic protein synthesis requires the action of not only the ribosome, mRNA and aminoacylated-tRNAs, but also series of soluble proteins that facilitate each step. Of the sixteen proteins grouped in “RNA metabolic process” (GO: 0016070), seven of them were overexpressed: elongation factor 1-alpha (EEF1A1, spot 302), WD repeat-containing protein1 (BRWD1, spot 309), the LIM domain of thyroid receptor-interacting protein 6 (TRIP6, spot 322) and heterogeneous nuclear ribonucleoprotein A/B-like (HNRNPAB, spot 664). There was a great increase of EEF1A1 expression and a decrease of proteins that catalyse the synthesis of glycyl-tRNA (GARS, spots 217 and 229) and seryl-tRNA (SARS, spot 482). The change in the former protein could be responsible for the enzymatic delivery of aminoacyl tRNAs to the ribosome^52^, and all these changes indicate the activation of protein synthesis machinery during myotube formation. Moreover, three proteins related to the translation process were overexpressed: cleavage stimulation factor subunit 1-like (CSTF1, spot 345), 40S ribosomal protein S12 (RPS12, spot 460) and ribosomal protein large P0-like protein (RPLP0, spot 625). RPS12 is responsible for the efficiency of selection of tRNA molecules during translation^53^ and RPL0 is a ribosomal protein that is a component of the 60S subunit. Meanwhile, four proteins related to the cell cycle were downregulated: protein arginine methyltransferase 5 (PRMT5, spot 244), which is involved in transcriptional repression and cell cycle progression^54^; ribonucleoside-diphosphate reductase large subunit (RRM1, spot 319), an enzyme essential for the production of deoxyribonucleotides prior to DNA synthesis in the S phase of dividing cells^55^ and two regulatory proteins from the 14-3-3 family, 14-3-3 protein epsilon-like (YWHAE, spot 680)and 14-3-3 protein beta/alpha-1-like (YWHAB, spots 682 and 890). These two highly conserved proteins are involved in signal transduction through linking mitogenic signalling and the cell cycle machinery.

In Fig. 9 highlights the main biological processes in the formation of post-mitotic plurinuclear myotubes, all of them related with synthesis processes. The main cluster, “cellular protein metabolic process” with a strong interaction of its proteins (0.79), grouped two clusters where most proteins overexpressed: “cellular amino acid metabolic process” (GO: 0006520), and “regulation of cellular aa metabolic process” (G: 0006521), this last with maximum interaction coefficient (1). It is noteworthy that aromatic amino acid (tyrosine and phenylalanine) metabolism seems to play a role in this phase, because three of the catabolic enzymes involved in “cellular amino acid metabolic process”: homogentisate 1, 2-dioxygenase (HGD, spot 335), 4-hydroxyphenylpyruvate dioxygenase-like (HPDL, spot 370) and dihydropteridine reductase (QDPR, spot 439), are upregulated. Moreover, Creatine kinase (spots 371 and 490), may be upregulated in relation to energy demand for protein synthesis. Other two overexpressed proteins in this phase, with a strong interaction coefficient (1) grouped in “pentose biosynthesis process (GO: 0019322)”. TKT (spot 293) and glucose-6-phosphate dehydrogenase (G6PD, spots 331 and 393) should signal that nucleotide and nucleic acid synthesis increased, together with high ribosomal activity, as we have indicated previously. Two highly overexpressed proteins are involved in microtubule formation, glyceraldehyde 3-phosphate dehydrogenase (GAPDH, spot360) and annexin (ANXA1, spot 411). Thus, the interactions of GAPDH with microtubules plays a structural role in the formation of the microtrabecular lattice^56^ and ANXA1 has been implicated in the regulation of the actin cytoskeleton, which is central to cell migration^57^.

The fusion of post-mitotic mononucleated myoblasts to form syncytial myofibres requires coordination of two processes: first, the remodelling of cell migration, adhesion, membrane fusion and the extra-cellular matrix^22^, and second, the maturation of both the contractile apparatus and entire organelles, peroxisomes and mitochondria^25^. In Fig. 10, two functional clusters related to these processes are shown, both of them with the highest local clustering coefficient, 1. In “muscle contraction” (GO: 0006936), actin (ACTA1, spot 43) and tropomyosin (TPM1, spot 655) overexpressed five and two times respectively. In the other cluster, “microtubule-based process”(GO:0007017), the downregulation observed of tubulin isoforms (TUBA8, spots 55 and 58, and TUBB, spots 85 and 609) and of the microtubule-associated protein RP/EB family (MAPRE1, spot 762) support the idea that the assembly process is over after around 12 days of culture. Thus, the cytoplasmic microtubule nucleation and the elongation processes promoted by MAPRE^58^ are finished. Moreover, the strong increase of two related proteins, keratin (KRT8, spot 115) stabilizing the epithelial cytoskeleton by providing resilience to mechanical stress^59^ and cofilin (CFL2, spot 453) as an ubiquitous actin-binding factor required for the reorganization of actin filaments, reinforced that myotube differentiation had finished.

## Conclusion

This study provides the first analysis of proteome profile changes during myogenesis in primary cultures of fish myocytes. The main changes in several of the most abundant proteins determine the importance of the cellular biological processes throughout myogenesis, and they correlate with the two phases, proliferation and differentiation, that are well established by the variations in gene expression, especially by myogenin. Thus, most proteins overexpressed at the beginning of the proliferation phase were related to cell cycle processes (e.g. ubiquitination, response to stress, and the pentose pathway), while a progressive increase of proteins involved in the processes of adhesion and reorganization of the cytoskeleton signalled the beginning of differentiation. The transition from proliferation to differentiation was also related to the overexpression of proteins involved in the glycolytic machinery and energy production, as well as in protein synthesis and turnover, and microtubule formation.

Although the number of proteins obtained via the 2D technique is much lower than that obtained by more sensitive techniques (such as shotgun or SILAC), the changes observed in the main cellular processes of both phases of myogenesis in fish coincide with those observed in mammals. Nevertheless, the peak of myogenin appears on days 8 and 9 in fish, whereas in mammals myotubes are already formed after 3 days. It is interesting to note that this expanded period of myocytes development in fish widens our possibilities of studying the mechanisms that regulate myogenesis. Also, the information provided may contribute to improvement of fish myocyte culture in the further attempt to establish fish cell myocyte lines.

## Methods

### Ethics statement

The Ethics and Animal Care Committee of the University of Barcelona, following the norms and procedures established by the EU (86/609/EU), Spain and the regional authorities, approved all animal handling procedures (permits CEEA 168/14 and DAAM 7749).

### Animals and experimental conditions

Gilthead sea bream (*Sparus aurata*) fingerlings obtained from Tinamenor Group (Pesués, Cantabria, Spain) were maintained in optimal conditions in the facilities of the Servei d’Estabulari of the Faculty of Biology, at the University of Barcelona. The fish were kept in 200 L fibreglass tanks with a closed-water flow circuit at a water temperature of 21 °C ± 1 °C, under a 12 h light/12 h dark photoperiod. They were fed *ad libitum* once daily with a commercial diet (Skretting España S.A., Burgos, Spain) and were fasted for 24 h prior to the isolation of muscle cells.

### Cell cultures

Five independent primary cultures of satellite cells were prepared from 70–80 animals (for each culture) with weights ranging from 5.3 to 6.1 g. The fish were killed by a sharp blow to the head, weighed, and immersed in 70% ethanol for 30 s to sterilize their external surfaces.

Satellite cell isolation was performed following the protocol previously described for gilthead sea bream^27^. To avoid contamination of fibroblasts as far as possible, the plates were washed quicky, as Froehlich *et al*.^60^ recommend, since fibroblasts are the first cells to attach to plates. Cells were seeded at a density of 1.5–2 × 10^6^ cells per well, in 6-well plastic plates (9.6 cm^2^/well), pre-treated with poly-L-lysine and laminin, and maintained at 18 °C in Dulbecco’s modified Eagle medium (DMEM) supplemented with 10% foetal bovine serum (FBS) and 1% antibiotic/antimycotic solution (AA). All plastic ware for tissue culture was obtained from Nunc (LabClinics, Barcelona, Spain) and all the reagents from Sigma–Aldrich (Tres Cantos, Spain) unless otherwise stated. Morphology was regularly observed to control the state of the cells. Cell development was followed, and images were taken at different times (days 4, 8 and 12) using an Axiovert 40 C inverted microscope (Carl Zeiss, Germany) coupled to a Canon digital camera.

Cells were maintained for 12 days, replacing the used medium with fresh, every 2 days. Sampling was performed on the 4th, 8th and 12th day of culture development as explained in what follows. For proteome analysis, the cells from 2 to 4 plates were harvested and combined. The medium was first removed, and the cells were washed twice with 2 mL of cold sterile PBS. Then the cells were harvested using lysis buffer (7 M urea, 2 M thiourea, 2% w/v CHAPS and 80 mM DTT) containing Protease Inhibitor Cocktail (Sigma-Aldrich, St. Louis, USA) by gently scraping. The homogenates were placed in rotary motion at 4 °C for about 1 hour and then centrifuged at 15 600 × g for 8 min at 4 °C. The resultant pellet was discarded, and the protein concentration of each supernatant fraction was determined following the Bradford method (using a commercial kit from Bio-Rad Laboratories, Madrid, Spain). The samples were stored at −80 °C until electrofocusing (IEF) and gel electrophoresis. For RNA analysis, samples were collected on the indicated days with 1 mL of TRI Reagent® solution (Applied Biosystems, Alcobendas, Spain), using cell-scrapers, and then stored at −80 °C.

### Myogenin analysis and quantitative real-time PCR (qPCR)

The concentration and purity of the total RNA obtained was determined using a NanoDrop ND2000 (Thermo Scientific, Alcobendas, Spain). To confirm their integrity, the samples were run in a 1% agarose gel and stained for visualization with SYBR® Safe DNA Gel Stain (Life Technologies, Alcobendas, Spain).

Following the previous described protocol^61^, two hundred nanograms of purified total RNA was treated with DNase I (Life Technologies, Alcobendas, Spain) for 15 min at room temperature to remove all genomic DNA. The DNase I was then inactivated by adding 1 µL of 25 nM EDTA and heating the sample to 65 °C for 10 min. After that, the RNA was reverse transcribed with the Transcriptor First Strand cDNA synthesis kit, following the manufacturer’s instructions (Roche, Sant Cugat del Valles, Spain) using anchored oligo (dT) 18 primers. qPCR reactions were carried out in a MyiQ Single-Color Real-Time PCR Detection System (Bio-Rad) following the guidelines for accurate analysis^62^. The amplifications were performed in a 20 µL reaction volume, containing 10 µL Q™ SYBR® Green Supermix (Bio-Rad, El Prat de Llobregat, Spain), 4 µL RNase/DNase-free water, 0.5 µL of forward and reverse primers (see Table 1) at 10 µM and 5 µL of template cDNA in a 96-well plate. Prior to analysis, a dilution curve was run with a pool of the samples to optimize the qPCR conditions. Two different genes were tested as controls: EF1α and RPL27a; due to better stability, the latter was selected as the reference gene. The primer sequences for RPL27a were obtained from Tiago *et al*.^63^, whereas for EF1α and myogenin, the primers were previously designed^19^. The expression level of myogenin gene was calculated relative to RPL27a gene expression, using the 2-ΔΔCT method^64^.

**Table 1:** Primers used for qPCR. Sequences, annealing temperature (Ta) and GenBank accession number.

Gene	Primer sequence (5′-3′)	Ta (°C)	Accession number	Reference
*Myogenin*	**F**: CAGAGGCTGCCCAAGGTCGAG **R**: GAGGTGCTGCCCGAACTGGGCTCG	68	EF462191	^19^
*EF1α*	**F**: CTTCAACGCTCAGGTCATCAT **R**: GCACAGCGAAACGACCAAGGGGA	60	AF184170	^19^
*RPL27a*	**F**: AAGAGGAACACAACTCACTGCCCCAC **R**: CTTGCCTTTGCCCAGAACTTTGTAG	68	AY188520	^63^

### 2D-PAGE

According to the previous protocol^65^ 300 μg of purified protein for each sample was dissolved into 450 μL of rehydration solution containing 7 M urea, 2 M thiourea, 2% w/v CHAPS, and 0.5% v/v IPG buffer pH 3–10 non-linear (Amersham Biosciences Europe, now GE Healthcare, Madrid, Spain), 80 mM DTT and 0.002% of bromophenol blue. The solution was then loaded onto 24 cm, pH 3–10 non-linear IPG strips. Isoelectric focusing was performed using an IPGphor instrument (Amersham Biosciences), following the manufacturer’s instructions (active rehydration at 50 V for 12 h, followed by a linear gradient from 500 to 8000 V at 68000 V/h). Focused strips were equilibrated in two steps as follows: 15 min with equilibration buffer I (65 mM DTT, 50 mM Tris-HCl, 6 M urea, 30% glycerol, 2% SDS, bromophenol blue) and then 15 min with equilibration buffer II (135 mM iodoacetamide, 50 mM Tris-HCl, 6 M urea, 30% glycerol, 2% SDS, bromophenol blue). The equilibrated strips were applied directly onto 12.5% polyacrylamide gels, sealed with 0.5% w/v agarose, and separated at a constant voltage of 50 V for 30 min followed by 200 V for about 6 h, until the blue dye reached the bottom, on an Ettan DALT II system (Amersham Biosciences, Stockholm, Sweden). The resolved proteins were fixed for 1 h in 40% v/v methanol containing 10% v/v acetic acid and stained overnight using colloidal Coomassie Blue G-250. The gel staining was removed by washing steps using distilled water, until optimum visualization was achieved.

### Protein visualization and image analysis

Coomassie blue gels were scanned in a calibrated Image Scanner III densitometer (GE Healthcare, Barcelona, Spain), and digital images captured at a resolution of 400 dpi in grey-scale mode using the Labscan 6.0 software (GE Healthcare, Barcelona, Spain) were saved as uncompressed TIFF files. Gels from five independent primary cell cultures were analysed using the software package Image Master 2D version 6.01 (GE Healthcare, Barcelona, Spain), which can be used to detect and obtain normalized volume values of the protein spots present on the gels. This detection was performed by the automated routines in the software, combined with manual editing to remove artefacts. The background was removed and normalized volumes were calculated as follows: we divided the volume of each spot by the total volume of all the spots marked in the gel.

### Gel image statistics

Matched spots were analysed by comparing the relative abundances between day 4 gels and day 8 gels (proliferation phase) and between day 8 gels and day 12 gels (differentiation phase). The differently expressed spots were obtained by unpaired sample t-test (SPSS v.16; Chicago, IL, USA). The Shapiro-Wilk test was previously used to ensure the normal distribution of the data, and the equality of variances was determined by Levene’s statistical test. The candidate spots selected for further MS analysis met the following criteria: ≥2-fold change in normalized volume (overexpressed) or <0.5-fold change in normalized volume (underexpressed) with significance (p < 0.05). We used the Perseus program in the MaxQuant software environment (http://www.maxquant.org) to generate and view hierarchical clustering and protein abundance heat maps.

### In-gel trypsin digestion and LC-MS/MS analysis

In-gel tryptic digestion was performed in an InvestigatorTM Progest automatic protein digestion system (Genomic Solutions). Protein spots of interest were excised with a razor blade from the 2-D gels, washed with ammonium bicarbonate (25 mM NH_4_HCO_3_) and acetonitrile (ACN). The sample was then immediately reduced (10 mM DTT; 30 min, 56 °C) and alkylated (55 mM iodoacetamide; 21 °C, 30 min, in the dark). Afterwards, the sample was digested with porcine trypsin (sequence grade modified trypsin, Promega; 80 ng trypsin/sample; 37 °C overnight for 16 h). Finally, the resulting peptide mixture was extracted and dried from the gel matrix with 10% formic acid (FA) and ACN, The extracts were then pooled and dried in a vacuum centrifuge.

Tryptic peptides were resuspended in 1% FA solution and an aliquot was injected for their chromatographic separation. Peptides were trapped in a Symmetry C18TM trap column (5 μm 180 μm × 20 mm; Waters, Madrid, Spain), and separated using a C18 reverse-phase capillary column (75 μm Oi, 25 cm, nanoAcquity, 1.7 μm BEH column; Waters). The gradient used for the elution of the peptides was 1% to 35% B in 30 minutes, followed by gradient from 35% to 50% in 5 minutes + 50–85% B in 2 min (A: 0.1% FA; B: 100% ACN, 0.1% FA), with a 250 nL/min flow rate. LC-MS coupling was performed with the AdvionTriversaNanomate (AdvionBioSciences, Ithaca, NY, USA) as the nanoESI source performing nanoelectrospray through chip technology. The Nanomate was attached to an LTQ-FT Ultra mass spectrometer (Thermo Fisher Scientific, Waltham, MA, USA) and operated at a spray voltage of 1.7 kV and a delivery pressure of 0.5 psi in positive mode. The mass spectrometer was operated in a survey data-dependent acquisition mode. Survey MS scans were acquired in the FT with a resolution of 100,000 FWHM at 400 m/z. Up to six of the most intense ions per scan were selected for fragmentation using collision-induced dissociation (CID) in the linear ion trap. Singly charged precursors were not selected for fragmentation. Target ions already selected for MS/MS were dynamically excluded for 30 s. the minimal signal required to trigger the MS to MS/MS switch was set to 1,000 and the activation Q was 0.250. The ion count target value was 1,000,000 for the survey scan and 50,000 for the MS/MS scan. Data were acquired with Thermo Xcalibur (v.2.1.0.1140; Thermo Electron, San Jose, CA, USA) software in raw data format and converted into mgf files by Proteome Discoverer v.1.3.0.339 (Thermo Fisher Scientific, Waltham, MA, USA) for further identification analysis.

### Protein identification

A database search was performed using the Mascot search engine and Thermo Proteome Discover (v.1.3.0.339) on both a database comprising all *Actinopterygii* entries in the NCBInr public database (378,755 proteins) and common laboratory contaminants. Both the target and a decoy database were searched to obtain a false discovery rate (FDR), and thus estimate the number of incorrect peptide-spectrum matches that exceed the desired threshold. We used the following parameters for the searches^65^: 2 missed cleavages, carbamidomethyl of cysteine as fixed modifications, and oxidation of methionine and pyro-Glu (N-term glutamine) as variable modifications. Peptide tolerance was 10 ppm and 0.6 Da for MS and MS/MS spectra, respectively. An automatic decoy database was included in the search of the latter data. Protein identifications were accepted when they were established at >95% probability. However, the protein identification with the highest score, discarding contaminants, was selected in the case of redundant protein identifications. Enrichment analyses of Gene Ontology (GO) annotation terms for biological processes were performed using the Batch-Genes tool produced by GOEAST (http://omicslab.genetics.ac.cn/GOEAST/). The Search Tool for the retrieval of Interacting Genes/Proteins (STRING program v10.5^66^) from *Homo sapiens* (id: 9606) was used to create protein networks of the differentially abundant proteins for each phase. The enrichment tests from the STRING software are used for a variety of classification systems, including Gene Ontology. The selected stat indicators were the “clustering coefficients” and the “PPI enrichment *p*-value”, which correspond to a measure of how connected the nodes in the network are, and the “count in gene set” indicating the number of proteins included, together with their “false discovery rate”.

Heat maps were generated from median z-scores of the differentially abundant proteins using agglomerative hierarchical cluster of Z-score transformed intensity data (Perseus program).

## Supplementary information

Supplementary Information

Dataset 1

## Data Availability

All data generated or analysed during this study are included in this published article (and its Supplementary Information Files).
