# Supplementary material for: Proteomic characterization of primary cultured myocytes in a fish model at different myogenesis stages
Source: Sci Rep. 2019 Oct 1;9:14126. doi: 10.1038/s41598-019-50651-w (PMC6773717; doi:10.1038/s41598-019-50651-w)
Supplement: Supplementary file 1 — Supplementary Information [file 41598_2019_50651_MOESM1_ESM.pdf]

**Title:** Proteomic characterization of primary cultured myocytes in a fish model at different myogenesis stages.

**Authors:** A. F. Millan-Cubillo, M. Martin-Perez, A. Ibarz, J. Fernandez-Borras,
J. Gutiérrez and J. Blasco

**Table S1. Identification of differentially expressed proteins spots during proliferation development stage in a primary muscle cell culture of gilthead sea bream**

| [a]SPOT | accession no. | [b]protein name | Species | [c]Symbol | Theorical Kda/pI | Observed Kda/pI | [e]score | [f]Peptides (P.Uniques) | [g]SC (%) | [h]FC | [i]p-value |
|---|---|---|---|---|---|---|---|---|---|---|---|
| **[d]Cytoeskeleton GO:0005856** | | | | | | | | | | | |
| 59 | gi\|348524070 | plastin 2 | *O. niloticus* | LCP1 | 69.1/5.3 | 73.0/4.7 | 969 | 14 (5) | 40 | 18.0 | 0.048 |
| 162 | gi\|317419458 | Vinculin | *D. labrax* | VCL | 118.0/5.8 | 130.0/4.9 | 828 | 14 (14) | 17 | 6.3 | 0.047 |
| 168 | gi\|317419458 | Vinculin | *D. labrax* | VCL | 118.0/5.8 | 128.0/5.1 | 2107 | 39 (11) | 37 | 8.1 | 0.005 |
| 169 | gi\|317419458 | Vinculin | *D. labrax* | VCL | 118.0/5.8 | 128.0/5.2 | 4018 | 66 (20) | 44 | 0.1 | 0.020 |
| 170 | gi\|317419458 | Vinculin | *D. labrax* | VCL | 118.0/5.8 | 142.0/5.0 | 3312 | 58 (5) | 45 | 8.5 | 0.062 |
| 172 | gi\|317419458 | Vinculin | *D. labrax* | VCL | 118.0/5.8 | 147.0/5.3 | 3212 | 65 (16) | 45 | 0.1 | 0.026 |
| 178 | gi\|432869188 | myosin heavy chain, fast skeletal muscle-like | *O. latipes* | MYH1 | 200.7/5.7 | 159.0/5.7 | 2007 | 50 (3) | 23 | 0.3 | 0.067 |
| 264 | gi\|339896073 | fast skeletal muscle myosin heavy chain isoform 1 | *S. chuatsí* | MYH1 | 221.4/5.8 | 107.0/5.1 | 2307 | 52 (2) | 24 | 0.1 | 0.007 |
| 364 | gi\|259155224 | adapter molecule crk | *S. salar* | CRK | 33.5/5.8 | 37.0/5.2 | 439 | 8 (6) | 26 | 0.5 | 0.053 |
| 758 | gi\|348507133 | PDZ and LIM domain protein 1-like | *O. niloticus* | PDLIM1 | 35.1/6.4 | 35.0/7.4 | 350 | 5 (2) | 16 | 0.4 | 0.054 |
| 336 | gi\|224796285 | type I keratin-like protein | *S. aurata* | KRTAP1-1 | 35.5/5.0 | 42.0/5.2 | 2153 | 30 (26) | 89 | 0.4 | 0.016 |
| **Others** | | | | | | | | | | | |
| 897 | gi\|410911576 | four and a half LIM domains protein 3-like | *T. rubripes* | FHL3 | 31.3/6.6 | 33.0/6.9 | 123 | 4 (4) | 9 | 0.1 | 0.052 |
| 566 | gi\|54261795 | tubulin, alpha 8 like 3 | *D. rerio* | TUBA8 | 50.0/5.1 | 53.0/5.9 | 439 | 15 (2) | 42 | 0.1 | 0.001 |
| 173 | gi\|499029532 | collagen alpha-1(VI) chain-like | *M. zebra* | COL6A1 | 106.8/5.8 | 150.0/5.5 | 141 | 2 (2) | 2 | 9.2 | 0.051 |
| **Cellular metabolic process (GO:0044237)** | | | | | | | | | | | |
| **[d] Aminoacyl-tRNA ligase activity GO:0004812** | | | | | | | | | | | |
| 161 | gi\|432862331 | alanine-tRNA ligase, cytoplasmic-like | *O. latipes* | AARS | 107.1/5.4 | 130.0/4.8 | 673 | 12 (2) | 9 | 5.2 | 0.010 |
| 537 | gi\|410912580 | cysteine--tRNA ligase, cytoplasmic-like | *T. rubripes* | CARS | 93.3/6.1 | 92.0/6.5 | 719 | 18 (6) | 18 | 2.8 | 0.044 |
| 564 | gi\|47215025 | aspartyl-tRNA synthetase | *T. nigroviridis* | DARS | 60.4/6.3 | 66.0/7.2 | 90 | 5 (5) | 11 | 0.3 | 0.003 |
| **Proteosome-mediated Ubiquitin-dependent protein catabolic process GO:0043161** | | | | | | | | | | | |
| 153 | gi\|409712094 | 94 kDa glucose-regulated protein | *S. aurata* | HSP90B1 | 92.7/4.8 | 93.0/4.7 | 2142 | 37 (37) | 38 | 7.7 | 0.016 |
| 176 | gi\|348529918 | phospholipase A-2-activating protein | *O. niloticus* | PLAA | 86.4/5.9 | 105.0/5.2 | 231 | 6 (6) | 8 | 2.8 | 0.009 |
| 192 | gi\|185132242 | valosin containing protein | *O. mykiss* | VCP | 83.0/5.3 | 74.0/5.1 | 283 | 5 (5) | 9 | 0.2 | 0.032 |
| 64 | gi\|499041071 | 26S protease regulatory subunit 6B-like isoform X2 | *M. zebra* | PSMC4 | 45.8/5.4 | 51.0/5.1 | 990 | 19 (19) | 50 | 0.4 | 0.001 |
| 901 | gi\|432860199 | proteasome subunit alpha type-1-like | *O. latipes* | PSMA1 | 29.3/6.5 | 28.0/7.4 | 720 | 12 (12) | 48 | 0.3 | 0.005 |
| 147 | gi\|409712094 | 94 kDa glucose-regulated protein | *S. aurata* | HSP90B1 | 92.7/4.8 | 117.0/4.4 | 666 | 24 (23) | 24 | 0.1 | 0.041 |
| **Generation of precursor metabolites and energy GO:0006091** | | | | | | | | | | | |
| 702 | gi\|410899691 | transketolase-like protein 2-like isoform 3 | *T. rubripes* | TKTL2 | 63.7/7.2 | 57.0/7.8 | 303 | 3 (3) | 11 | 0.3 | 0.018 |
| 667 | gi\|348521484 | transketolase | *O. niloticus* | TKT | 67.8/6.5 | 70.0/7.4 | 115 | 3 (3) | 6 | 0.4 | 0.010 |
| 515 | gi\|17433114 | L-lactate dehydrogenase A chain | *H. antarcticus* | LDHA | 36.2/7.2 | 32.0/4.4 | 92 | 2 (2) | 7 | 0.5 | 0.059 |
| 881 | gi\|432861321 | pyruvate kinase muscle isozyme-like isoform 2 | *O. latipes* | PKM | 50.4/7.8 | 30.0/5.6 | 219 | 3 (3) | 9 | 0.2 | 0.008 |
| 601 | gi\|198285519 | electron-transfer-flavoprotein alpha polypeptide | *S. salar* | ETFA | 34.9/7.8 | 32.0/6.1 | 408 | 5 (5) | 27 | 0.4 | 0.002 |
| 654 | gi\|499026726 | glycogen phosphorylase, muscle form-like | *M. zebra* | PYGM | 97.2/7.1 | 98.0/6.9 | 321 | 7 (2) | 8 | 2.0 | 0.020 |
| 892 | gi\|410899354 | alpha-enolase-like isoform 1 | *T. rubripes* | ENO1 | 46.9/6.6 | 53.0/6.9 | 1117 | 17 11) | 47 | 3.6 | 0.015 |
| 23 | gi\|348506725 | ATP synthase subunit beta, mitochondrial-like | *O. niloticus* | ATP5B | 55.1/5.2 | 55.0/4.1 | 1334 | 15 (15) | 35 | 2.4 | 0.009 |

| [a]SPOT | accession no. | [b]protein name | Species | [c]Symbol | Theorical Kda/pI | Observed Kda/pI | [e]score | [f]Peptides (P.Uniques) | [g]SC (%) | [h]FC | [i]p-value |
|---|---|---|---|---|---|---|---|---|---|---|---|
| **Others** | | | | | | | | | | | |
| 850 | gi\|384267662 | core binding factor beta | *C. idella* | CBFB | 22.1/6.2 | 17.0/5.7 | 370 | 5 (5) | 35 | 2.3 | 0.025 |
| 816 | gi\|380857385 | carbonyl reductase-like 20beta-hydroxysteroid dehydrogenase | *S. aurata* | CBR1 | 29.7/8.5 | 17.0/7.1 | 207 | 3 (3) | 14 | 3.1 | 0.015 |
| 257 | gi\|498973250 | programmed cell death 6-interacting protein-like isoform X1 | *M. zebra* | PDCD6IP | 97.6/6.0 | 110.0/5.8 | 1356 | 22 (11) | 26 | 2.8 | 0.042 |
| 324 | gi\|498990975 | synaptic vesicle membrane protein VAT-1 homolog | *M. zebra* | VAT1 | 53.5/6.8 | 40.0/5.4 | 498 | 18 (4) | 23 | 0.5 | 0.006 |
| 685 | gi\|410912454 | bifunctional purine biosynthesis protein PURH-like | *T. rubripes* | ATIC | 64.3/7.3 | 69.0/7.9 | 890 | 12 (3) | 24 | 0.3 | 0.018 |
| 666 | gi\|348521966 | far upstream element-binding protein 1 | *O. niloticus* | FUBP1 | 66.1/7.0 | 76.0/7.4 | 103 | 9 (9) | 18 | 0.4 | 0.003 |
| 422 | gi\|333411049 | acidic ribosomal phosphoprotein P0 | *S. lalandI* | RPLP0 | 17.4/6.8 | 24.0/4.1 | 174 | 2 (2) | 15 | 0.4 | 0.060 |
| 904 | gi\|333827865 | peroxiredoxin 1 | *S. aurata* | PRDX1 | 22.0/6.8 | 27.0/7.4 | 762 | 9 (8) | 34 | 0.4 | 0.000 |
| 905 | gi\|157929894 | natural killer cell enhancement factor | *E. coioides* | PRDX1 | 20.1/6.5 | 27.0/7.5 | 106 | 2 (2) | 12 | 0.5 | 0.013 |

**[a] number of spots analyzed by Orbitrap. [b] Proteins name derived from BLASTp sequence analysis. [c] Symbol of gene product from GeneCards v3.07. [d] Associated Gene Ontology term. [e] MASCOT score obtained > score corresponding to $p < 0.05$ (probability 95%). [f] Number of total peptides observed Analysis, in parentheses unique peptides from the LC-MS/MS analysis.[g] Coverage percentage of the peptide sequence homology. [h] Fold change D4 vs D8 (>0, up-regulated; <0, down-regulated) . [i] Student t-test (n = 5).**

**Table S2. Identification of differentially expressed proteins spots during differentiation development stage in a primary muscle cell culture of gilthead sea bream.**

| [a]SPOT | accession no. | [b]protein name | Species | [c]Symbol | theorical Kda/pI | Observed Kda/pI | [e]score | [f]Peptides (P.Uniques) | [g]SC (%) | [h]FC | [i]p-value |
|---|---|---|---|---|---|---|---|---|---|---|---|
| **[d] Protein folding GO: 0006457** | | | | | | | | | | | |
| 119 | gi\|355329972 | heat shock 70kDa protein 5 | *O. latipes* | HSPA5 | 72,1/5,1 | 81.0/4.2 | 983 | 15(3) | 30 | 3.0 | 0.001 |
| 174 | gi\|409712094 | 94 kDa glucose-regulated protein | *S. aurata* | HSP90B1 | 92,7/4,8 | 90.0/4.8 | 61 | 2 (2) | 2 | 2.0 | 0.037 |
| 114 | gi\|348512803 | endoplasmin-like | *O. niloticus* | HSP90B1 | 91,5/4,8 | 118.0/4.3 | 587 | 13(13) | 16 | 0.3 | 0.003 |
| 305 | gi\|47215719 | T-complex protein 1 subunit eta-like | *T. nigroviridis* | CCT7 | 59,3/6,3 | 72.0/7.5 | 6566 | 36(8) | 52 | 2.4 | 0.018 |
| 307 | gi\|71724948 | heat shock protein 60 | *L. crocea* | HSPD1 | 61,6/6,7 | 76.0/7.7 | 751 | 11 (8) | 19 | 2.7 | 0.009 |
| 82 | gi\|193788703 | protein disulfide-isomerase precursor | *D. rerio* | PDIA3 | 56,6/4,7 | 63.0/4.4 | 149 | 3(3) | 11 | 0.4 | 0.001 |
| 127 | gi\|348162167 | heat shock protein 90-beta | *L. crocea* | HSP90AB1 | 83,3/5,0 | 110.0/4.5 | 3523 | 42(42) | 49 | 0.2 | 0.05 |
| 128 | gi\|348162167 | heat shock protein 90-beta | *L. crocea* | HSP90AB1 | 83,3/5,0 | 102.0/4.4 | 3609 | 36 (4) | 44 | 0.2 | 0.039 |
| 108 | gi\|302030266 | heat shock protein 90 | *L. sanguineus* | HSP90AB1 | 83,1/5,0 | 99.0/3.7 | 190 | 5(5) | 10 | 0.5 | 0.009 |
| 186 | gi\|119692141 | glucose regulated protein 75 | S. aurata | HSPA9 | 68,7/5,7 | 75.0/5.5 | 5200 | 37 (21) | 56 | 0.3 | 0.039 |
| 232 | gi\|498962433 | PREDICTED: neutral alpha-glucosidase AB-like isoform X3 | M. zebra | GANAB | 105,7/5,7 | 141.0/6.1 | 721 | 14 (3) | 14 | 0.3 | 0.045 |
| 557 | gi\|410896388 | PREDICTED: 60 kDa heat shock protein, mitochondrial-like | *T. rubripes* | HSPD1 | 61,1/5,7 | 57.0/5.6 | 151 | 3 (3) | 7 | 0.5 | 0.037 |
| 779 | gi\|48526090 | 14 kDa apolipoprotein | *S. aurata* | APOA2 | 15,9/5,3 | 20.0/4.3 | 114 | 2 (2) | 27 | 3.0 | 0.029 |
| | | | | | | | | | | | |
| 537 | gi\|332380488 | tapasin precursor | *D. labrax* | TAPBP | 47,6/5,9 | 52.0/5.8 | 128 | 3(3) | 8 | 3.7 | 0.025 |
| **RNA metabolic process GO: 0016070** | | | | | | | | | | | |
| 302 | gi\|5923899 | elongation factor 1-alpha | *S. aurata* | EEF1A1 | 50,6/9,2 | 80.0/7.4 | 101 | 9 (4) | 31 | 6.7 | 0.006 |
| 309 | gi\|317419065 | WD repeat-containing protein 1 | *D. labrax* | BRWD1 | 66,6/6,4 | 81,0/7,7 | 731 | 10 (7) | 16 | 2.6 | 0.050 |
| 322 | gi\|348513605 | LIM domain of Thyroid receptor-interacting protein 6 | *O. niloticus* | TRIP6 | 61,7/7,3 | 94.0/7.6 | 115 | 7(7) | 18 | 2.5 | 0.050 |
| 664 | gi\|348535473 | PREDICTED: heterogeneous nuclear ribonucleoprotein A/B-like | *O. niloticus* | HNRNPAB | 36,8/5,1 | 42.0/4.2 | 300 | 6 (4) | 14 | 2.0 | 0.018 |
| 217 | gi\|317419952 | Glycyl-tRNA synthetase | D. labrax | GARS | 85,4/6,6 | 90.0/5.8 | 476 | 9 (3) | 14 | 0.4 | 0.001 |
| 229 | gi\|189536638 | glycyl-tRNA synthetase | *D. rerio* | GARS | 86,3/7,2 | 90.0/6.3 | 639 | 8(8) | 16 | 0.4 | 0.002 |
| 375 | gi\|348514662 | PREDICTED: alpha-enolase-like isoform 2 | O. niloticus | ENO1 | 42,5/6,7 | 46.0/7.1 | 305 | 7 (7) | 20 | 0.4 | 0.010 |
| 482 | gi\|226358659 | seryl-tRNA synthetase | H. nobilis | SARS | 22,7/5,9 | 22.0/6.3 | 225 | 2 (2) | 15 | 0.5 | 0.047 |
| 519 | gi\|410899354 | PREDICTED: alpha-enolase-like isoform 1 | *T. rubripes* | ENO1 | 46,9/6,6 | 45.0/6.0 | 2379 | 16 (16) | 49 | 0.5 | 0.049 |
| 526 | gi\|348514662 | PREDICTED: alpha-enolase-like isoform 2 | *O. niloticus* | ENO1 | 42,5/6,7 | 51.0/6.4 | 753 | 7 (7) | 26 | 0.5 | 0.051 |
| 552 | gi\|37590349 | Enolase 1, (alpha) | *D. rerio* | ENO1 | 47,0/6,6 | 54.0/5.8 | 2609 | 26(3) | 53 | 0.4 | 0.001 |
| 345 | gi\|348518026 | cleavage stimulation factor subunit 1-like | *O. niloticus* | CSTF1 | 48,3/6,6 | 62.0/7.3 | 181 | 6(6) | 21 | 3.3 | 0.007 |
| 460 | gi\|47224253 | 40S ribosomal protein S12 | *T. nigroviridis* | RPS12 | 14,4/7,2 | 70.0/7.7 | 321 | 7(7) | 48 | 3.8 | 0.001 |
| 538 | gi\|499051022 | PREDICTED: eukaryotic initiation factor 4A-II-like isoform X3 | M. zebra | EIF4A2 | 44,4/5,7 | 52.0/6.0 | 146 | 3 (3) | 8 | 2.8 | 0.015 |
| 625 | gi\|48476454 | ribosomal protein large P0-like protein | *S. aurata* | RPLP0 | 33,9/6,0 | 37.0/5.4 | 683 | 19(19) | 58 | 3.7 | 0.052 |
| 219 | gi\|432874742 | PREDICTED: splicing factor 3A subunit 1-like | *O. latipes* | SF3A1 | 88,1/5,6 | 94.0/5.7 | 109 | 2 (2) | 4 | 0.4 | 0.012 |
| 244 | gi\|167651000 | PRMT5 (protein arginine methyltransferase 5) | *O. latipes* | PRMT5 | 71,8/6,1 | 73.0/6.4 | 388 | 8 (1) | 11 | 0.3 | 0.042 |
| 682 | gi\|432865702 | PREDICTED: 14-3-3 protein beta/alpha-1-like | *O. latipes* | YWHAB | 27,6/4,7 | 25.0/4.0 | 357 | 8 (8) | 35 | 0.3 | 0.009 |
| 890 | gi\|432865702 | PREDICTED: 14-3-3 protein beta/alpha-1-like | *O. latipes* | YWHAB | 27,6/4,7 | 25.0/4.3 | 938 | 11 (6) | 48 | 0.2 | 0.008 |
| 680 | gi\|348532526 | 14-3-3 protein epsilon-like | *O. niloticus* | YWHAE | 29,1/4,8 | 28.0/4.2 | 1212 | 15 (4) | 56 | 0.1 | 0.000 |
| | | | | | | | | | | | |
| 511 | gi\|498925046 | PREDICTED: annexin A3-like | M. zebra | ANXA3 | 37,2/5,7 | 38.0/5.8 | 177 | 3 (3) | 10 | 0.4 | 0.008 |
| 449 | gi\|169643691 | hypoxanthine phosphoribosyltransferase | *S. senegalensis* | HPRT1 | 25,1/6,4 | 27.0/7.4 | 366 | 8(8) | 25 | 2.2 | 0.009 |
| 319 | gi\|213512716 | Ribonucleoside-diphosphate reductase large subunit | *S. salar* | RRM1 | 89,7/6,6 | 100.0/7.3 | 409 | 18 (6) | 25 | 0.4 | 0.001 |

| [a]SPOT | accession no. | [b]protein name | Species | [c]Symbol | theorical Kda/pI | Observed Kda/pI | [e]score | [f]Peptides (P.Uniques) | [g]SC (%) | [h]FC | [i]p-value |
|---|---|---|---|---|---|---|---|---|---|---|---|
| **Celular protein metabolic process GO: 0044267** | | | | | | | | | | | |
| 360 | gi\|15146358 | glyceraldehyde 3-phosphate dehydrogenase | *P. major* | GAPDH | 36,0/6,8 | 40./7.4 | 2278 | 26(21) | 62 | 2.4 | 0.029 |
| 411 | gi\|157278387 | annexin max3 | *O. latipes* | ANXA1 | 37,4/7,6 | 37.0/7.2 | 716 | 6(4) | 19 | 2.5 | 0.011 |
| 41 | gi\|34785428 | Protein arginine methyltransferase 1 | *D. rerio* | PRMT1 | 39,3/5,7 | 42.0/4.9 | 241 | 3 (3) | 12 | 0.3 | 0.043 |
| 233 | gi\|292625925 | lon protease homolog mitochondrial | *D. rerio* | LONP1 | 107,7/6,4 | 141.0/6.2 | 686 | 19 (4) | 20 | 0.2 | 0.023 |
| 265 | gi\|348508094 | protein-glutamine gamma-glutamyltransferase 2-like | *O. niloticus* | TGM2 | 75,2/6,8 | 101.0/6.9 | 252 | 7(3) | 10 | 0.4 | 0.006 |
| 402 | gi\|116488168 | eukaryotic translation elongation factor 2 | *S. maximus* | EEF2 | 23,7/7,0 | 39.0/6.7 | 440 | 7 (4) | 31 | 0.5 | 0.047 |
| 407 | gi\|15146358 | glyceraldehyde 3-phosphate dehydrogenase | *P. major* | GAPDH | 36,0/6,8 | 38.0/7.0 | 948 | 15 (15) | 49 | 0.2 | 0.003 |
| 817 | gi\|409712148 | Cu-Zn superoxide dismutase | *S. aurata* | SOD1 | 15,8/6,2 | 90.0/5.9 | 406 | 5 (5) | 52 | 0.4 | 0.044 |
| 871 | gi\|15718387 | gelatinase | *P. olivaceus* | MMP2 | 74,5/5,1 | 72.0/4.4 | 172 | 4(2) | 5 | 0.2 | 0.054 |
| 65 | gi\|348506725 | PREDICTED: ATP synthase subunit beta, mitochondrial-like | *O. niloticus* | ATP5B | 55,1/5,2 | 56.0/4.2 | 4121 | 27 (27) | 67 | 0.5 | 0.026 |
| 89 | gi\|198285477 | ATP synthase H+ transporting mitochondrial F1 complex beta | *S. salar* | ATP5B | 52,9/5,0 | 56.0/4.2 | 113 | 6(6) | 17 | 0.4 | 0.000 |
| **Celular amino acid metabolic process GO: 0006520** | | | | | | | | | | | |
| 335 | gi\|317419843 | Homogentisate 1,2-dioxygenase | *D. labrax* | HGD | 49,7/6,5 | 58.0/7.5 | 435 | 9(8) | 19 | 2.7 | 0.048 |
| 370 | gi\|348516106 | 4-hydroxyphenylpyruvate dioxygenase-like | *O. niloticus* | HPDL | 44,8/6,3 | 47.0/7.2 | 5355 | 10(6) | 30 | 2.3 | 0.010 |
| 371 | gi\|255502901 | muscle-type creatine kinase | *S. chuatsi* | CKM | 42,9/6,8 | 47.0/7.2 | 461 | 11(4) | 30 | 2.0 | 0.053 |
| 429 | gi\|110351012 | S-adenosylhomocysteine hydrolase-like protein | *P promelas* | AHCY | 31,6/7,1 | 30.0/7.5 | 90 | 2 (2) | 10 | 2.0 | 0.012 |
| 439 | gi\|47227499 | dihydropteridine reductase | *T. nigroviridis* | QDPR | 24,4/7,4 | 24.0/7.4 | 7894 | 6(6) | 25 | 3.9 | 0.031 |
| 490 | gi\|156972295 | creatine kinase isoform a | *H. hippoglossus* | CKM | 27,2/7,3 | 27.0/7.2 | 213 | 5 (1) | 20 | 2.0 | 0.051 |
| 581 | gi\|348515507 | argininosuccinate synthase-like | *O. niloticus* | ASS1 | 46,5/6,8 | 52.0/5.5 | 98 | 4(4) | 9 | 4.5 | 0.017 |
| **Regulation of celular amino acid metabolic process GO: 0006521** | | | | | | | | | | | |
| 354 | gi\|213513780 | 26S proteasome non-ATPase regulatory subunit 7 like 1 | *S. salar* | PSMD7 | 37,2/6,6 | 42.0/7.5 | 797 | 12(1) | 41 | 2.5 | 0.012 |
| 422 | gi\|432945585 | PREDICTED: 26S protease regulatory subunit 10B-like | *O. latipes* | PSMC6 | 42,9/7,8 | 33.0/7.7 | 632 | 14 (14) | 41 | 3.7 | 0.048 |
| 757 | gi\|410898722 | PREDICTED: proteasome subunit alpha type-3-like | *T. rubripes* | PSMA3 | 28,4/5,2 | 27.0/5.0 | 278 | 6 (6) | 28 | 2.0 | 0.012 |
| 685 | gi\|229367530 | Proteasome subunit alpha type-5 | *A. fimbria* | PSMA5 | 26,4/4,8 | 25.0/4.0 | 4279 | 16 (4) | 65 | 0.2 | 0.019 |
| **Pentose byosinthesis process GO: 0019322** | | | | | | | | | | | |
| 293 | gi\|348521484 | PREDICTED: transketolase | *O. niloticus* | TKT | 67,8/6,5 | 76.0/7.2 | 148 | 6 (6) | 12 | 2.1 | 0.054 |
| 331 | gi\|2734869 | glucose-6-phosphate dehydrogenase | *T. rubripes* | G6PD | 58,8/6,9 | 66.0/7.6 | 923 | 14 (2) | 26 | 2.2 | 0.039 |
| 393 | gi\|58803037 | hepatic glucose-6-phosphate dehydrogenase | R. sarba | G6PD | 58,5/7,7 | 66.0/7.0 | 4857 | 39(26) | 57 | 2.0 | 0.054 |
| **Muscle contraction GO: 0006936** | | | | | | | | | | | |
| 43 | gi\|224712556 | muscle actin type 1 | *H. mylodon* | ACTA1 | 41,9/5,3 | 44.0/4.9 | 331 | 10 (2) | 34 | 4.5 | 0.021 |
| 11 | gi\|1351868 | alpha skeletal muscle | *C. auratus* | ACTA1 | 41,9/5,4 | 48.0/4.7 | 1415 | 17 (9) | 51 | 0.4 | 0.035 |
| 188 | gi\|317419458 | Vinculin | *D. labrax* | VCL | 118,0/5,8 | 118.0/5.0 | 3435 | 48(5) | 45 | 6.8 | 0.039 |
| 197 | gi\|432924372 | Vinculin-like | *O. latipes* | VCL | 116,7/6,3 | 109.0/5.4 | 188 | 5 (5) | 5 | 0.4 | 0.015 |
| 236 | gi\|317419458 | Vinculin | *D. labrax* | VCL | 118,0/5,8 | 163.0/6.2 | 4686 | 48(48) | 47 | 0.2 | 0.016 |
| 672 | gi\|499003027 | PREDICTED: tropomyosin alpha-4 chain-like isoform X5 | *M. zebra* | TPM4 | 28,1/4,7 | 21.0/4.0 | 405 | 8 (7) | 29 | 0.2 | 0.005 |
| 567 | gi\|348515631 | PREDICTED: desmin-like | *O. niloticus* | DES | 54,1/5,4 | 59.0/4.3 | 507 | 9 (1) | 24 | 0.4 | 0.021 |
| 655 | gi\|60390740 | Tropomyosin alpha-1 chain | P. argentata | TPM1 | 32,7/4,7 | 36.0/4.1 | 4075 | 50 (7) | 90 | 2.1 | 0.001 |
| **Microtubule based process GO: 0007017** | | | | | | | | | | | |
| 55 | gi\|291190552 | tubulin, alpha 8 like 3-1 | *S. salar* | TUBA8 | 50,1/5,1 | 61.0/4.8 | 1086 | 17 (4) | 56 | 0.4 | 0.003 |
| 85 | gi\|197632605 | tubulin, alpha 8 like 3-2 | *S. salar* | TUBA8 | 50,0/5,1 | 61.0/4.3 | 347 | 9 (9) | 33 | 0.3 | 0.053 |
| 609 | gi\|47215416 | unnamed protein product (tubulin beta chain) | *T. nigroviridis* | TUBB | 42,8/5,5 | 34.0/5.7 | 325 | 6 (3) | 17 | 0.4 | 0.040 |

| | | | | | | | | | | | |
|---|---|---|---|---|---|---|---|---|---|---|---|
| 58 | gi\|348535178 | tubulin beta-1 chain-like | *O. niloticus* | TUBB | 49,7/4,9 | 59.0/4.5 | 8392 | 37(6) | 77 | 0.2 | 0.049 |
| 130 | gi\|348522746 | lamin-B2-like | *O. niloticus* | LMNB2 | 65,1/5,2 | 74.0/4.4 | 76 | 3(3) | 5 | 0.2 | 0.002 |
| 115 | gi\|317418883 | Keratin, type II cytoskeletal 8 | *D. labrax* | KRT8 | 61,7/5,1 | 82.0/4.2 | 3064 | 29(12) | 32 | 3.9 | 0.005 |
| 453 | gi\|328677135 | cofilin 2 | *E. bruneus* | CFL2 | 18,9/7,4 | 17.0/7.5 | 722 | 12(12) | 36 | 2.1 | 0.040 |
| 762 | gi\|348517001 | microtubule-associated protein RP/EB family member 1-like | *O. niloticus* | MAPRE1 | 29,3/5,2 | 28.0/4.8 | 902 | 14 (9) | 35 | 0.1 | 0.023 |
| **Others** | | | | | | | | | | | |
| 171 | gi\|410931724 | PREDICTED: major vault protein-like | *T. rubripes* | MVP | 98,2/5,6 | 88.0/5.0 | 234 | 6 (6) | 9 | 2.4 | 0.027 |
| 187 | gi\|291190570 | major vault protein | *S. salar* | MVP | 98,0/5,7 | 131.0/5.0 | 52 | 3(2) | 4 | 0.2 | 0.031 |
| 352 | gi\|348504238 | PREDICTED:short-chain specific acyl-CoA dehydrogenase, mitochondrial-like | *O. niloticus* | ACADS | 43,7/7,7 | 45.0/7.7 | 73 | 2 (2) | 5 | 3.6 | 0.016 |
| 776 | gi\|317419726 | Isopentenyl-diphosphate Delta-isomerase 1 | *D. labrax* | IDI1 | 26,6/5,6 | 24.0/5.9 | 269 | 5 (5) | 20 | 0.2 | 0.027 |
| 211 | gi\|498973250 | PREDICTED: programmed cell death 6-interacting protein-like isoform X1 | M. zebra | PDCD6IP | 97,6/6,0 | 114.0/5.8 | 1591 | 28 (11) | 30 | 0.3 | 0.017 |
| 84 | gi\|47220951 | unnamed protein product (nucleolin) | *T. nigroviridis* | NCL | 64,5/4,7 | 68.0/4.2 | 272 | 5 (3) | 8 | 0.5 | 0.017 |
| 185 | gi\|47214138 | tetratricopeptide repeat domain 34 | *T. nigroviridis* | TTC34 | 69,5/6,4 | 73.0/5.4 | 1336 | 20 (9) | 37 | 2.3 | 0.000 |
| 426 | gi\|348527950 | voltage-dependent anion-selective channel protein 1-like | *O. niloticus* | VDAC1 | 30,4/7,5 | 29.0/7.8 | 15005 | 30(2) | 64 | 3.1 | 0.005 |
| 448 | gi\|348529230 | V-type proton ATPase subunit E 1-like isoform 2 | *O. niloticus* | ATP6V1E1 | 23,5/9,0 | 27.0/7.5 | 90 | 4 (4) | 21 | 3.8 | 0.015 |
| 42 | gi\|224796285 | type I keratin-like protein | *S. aurata* | KRT6B | 35,5/5,0 | 44.0/4.9 | 2153 | 30 (26) | 89 | 2.4 | 0.008 |
| 766 | gi\|47218643 | unnamed protein product (grancalcin) | *T. nigroviridis* | GCA | 25,7/5,2 | 22.0/5.3 | 105 | 2 (2) | 5 | 0.3 | 0.028 |
| 900 | gi\|224796285 | type I keratin-like protein | *S. aurata* | KRT13 | 35,5/5,0 | 38.0/4.7 | 606 | 17 (9) | 49 | 0.5 | 0.062 |
| 221 | gi\|348537470 | moesin-like | *O. niloticus* | MSN | 67,8/5,9 | 76.0/6.2 | 3064 | 26(8) | 42 | 0.2 | 0.046 |
| 645 | gi\|348509649 | reticulocalbin-1-like | *O. niloticus* | RCN1 | 41,2/4,7 | 44.0/3.9 | 190 | 7(7) | 16 | 0.4 | 0.001 |

**[a] number of spots analyzed by Orbitrap. [b] Proteins name derived from BLASTp sequence analysis. [c] Symbol of gene product from GeneCards v3.07. [d] Associated Gene Ontology term. [e] MASCOT score obtained > score corresponding to p < 0.05 (probability 95%). [f] Number of total peptides observed Analysis, in parentheses unique peptides from the LC-MS/MS analysis. [g] Coverage percentage of the peptide sequence homology. [h] Fold change D8 vs D12 (>0, up-regulated; <0, down-regulated) . [i] Student t-test (n = 5).**

**Table S3.** Network stats and functional enrichments of proteins differentially expressed at proliferation stage.

| | Fig 6A | Fig 7 | Fig 7 | Fig 7 | Fig 7 |
|---|---|---|---|---|---|
| Interactome networks | Global Interactome Proliferation | Cytoeskeleton | Aminoacyl-tRNA ligase activity | Proteosome-mediated ubiquitin-dependent protein catabolic process | Generation of precursor metabolites and energy |
| Network stats[1] | | | | | |
| *Average local clustering coefficient* | 0.556 | 0.521 | 0.667 | 0.833 | 0.805 |
| *PPI enrichment p-value* | 9.83e-05 | 3.96e-06 | 0.000217 | 5.04e-05 | 3.79e-11 |
| Functional enrichment[2] | | | | | |
| *Pathway ID* | | GO:0005856 | GO:0004812 | GO:0043161 | GO:0006091 |
| *Pathway description* | | Cytoeskeleton | Aminoacyl-tRNA ligase activity | Proteosome-mediated ubiquitin-dependent protein catabolic process | Generation of precursor metabolites and energy |
| *Count in gene set* | | 6 | 3 | 4 | 7 |
| *False discovery rate* | | 0.00804 | 1.02e-05 | 0.000866 | 3.54e-07 |

1- Network stats: Clustering coefficients correspond to a measure of how connected the nodes in the network are. Highly connected networks have high values (between 0 and 1). Low PPI (protein-protein interaction) enrichment p-values indicate that the nodes are not random and that the observed number of edges is significant. Both indices are obtained from data analysis by STRING Program v10.5 (NCBI taxonomy id: 9606, current organism: *Homo sapiens*).

2- Functional enrichments of each cluster (Figure 7) have been performed using the protein groups from Supplementary Table S1. Pathway ID and description matched with those reported in Table S1, being "count in gene set" the number of proteins included with their "false discovery rate".

Basic settings used for STRING analysis: "confidence" and all of the active interactions sources (textmining, experiments, databases, co-expression, neighborhood gene fusion and co-occurrence).

**Table S4.** Network stats and functional enrichments of proteins differentially expressed at differentiation stage.

| | Fig 6 B | Fig 8 | Fig 8 | Fig 9 | Fig 9 | Fig 9 | Fig 9 | Fig 9 | Fig 9 |
|---|---|---|---|---|---|---|---|---|---|
| Interactome networks | Global Interactome<br><br>Differentiation | Protein folding | RNA metabolic process | Cellular protein metabolic process | Cellular amino acid metabolic process | Regulation of cellular aa metabolic process | Pentose biosynthesis process | Muscle contraction | Microtubule based process |
| Network stats[1] | | | | | | | | | |
| *Average local clustering coefficient* | 0.539 | 0.813 | 0.678 | 0.792 | 0.333 | 1.000 | 1.000 | 1.000 | 1.000 |
| *PPI enrichment p-value* | <1.0e-16 | 1.65e-11 | 0.000128 | 0.00257 | 0.0533 | 2.08e-06 | 0.0229 | 3.66e-10 | 0.00439 |
| Functional enrichment[2] | | | | | | | | | |
| *Pathway ID* | | GO:0006457 | GO:001670 | GO:0044267 | GO:0006520 | GO:0006521 | GO:0019322 | GO:0006936 | GO:0007017 |
| *Pathway description* | | Cytoeskeleton | Aminoacyl-tRNA ligase activity | Proteosome-mediated ubiquitin-dependent protein catabolic process | Generation of precursor metabolites and energy | | | | |
| *Count in gene set* | | 8 | 13 | 11 | 5 | 4 | 2 | 5 | 2 |
| *False discovery rate* | | 4.08e-11 | 3.51e-05 | 4.95e-05 | 6.75e-05 | 1.41e-07 | 0.000395 | 3.85e-07 | 0.0145 |

1- Network stats: Clustering coefficients correspond to a measure of how connected the nodes in the network are. Highly connected networks have high values (between 0 and 1). Low PPI (protein-protein interaction) enrichment p-values indicate that the nodes are not random and that the observed number of edges is significant. Both indices are obtained from data analysis by STRING Program v10.5 (NCBI taxonomy id: 9606, current organism: *Homo sapiens*).

2- Functional enrichments of each cluster (Figure 7) have been performed using the protein groups from Supplementary Table S2. Pathway ID and description matched with those reported in Table S2, being "count in gene set" the number of proteins included with their "false discovery rate".

Basic settings used for STRING analysis: "confidence" and all of the active interactions sources (textmining, experiments, databases, co-expression, neighborhood gene fusion and co-occurrence).

**Figure S1:** Reproducibility of 2D-gels: C1D4 to C5D12 ordered as in Table S5.

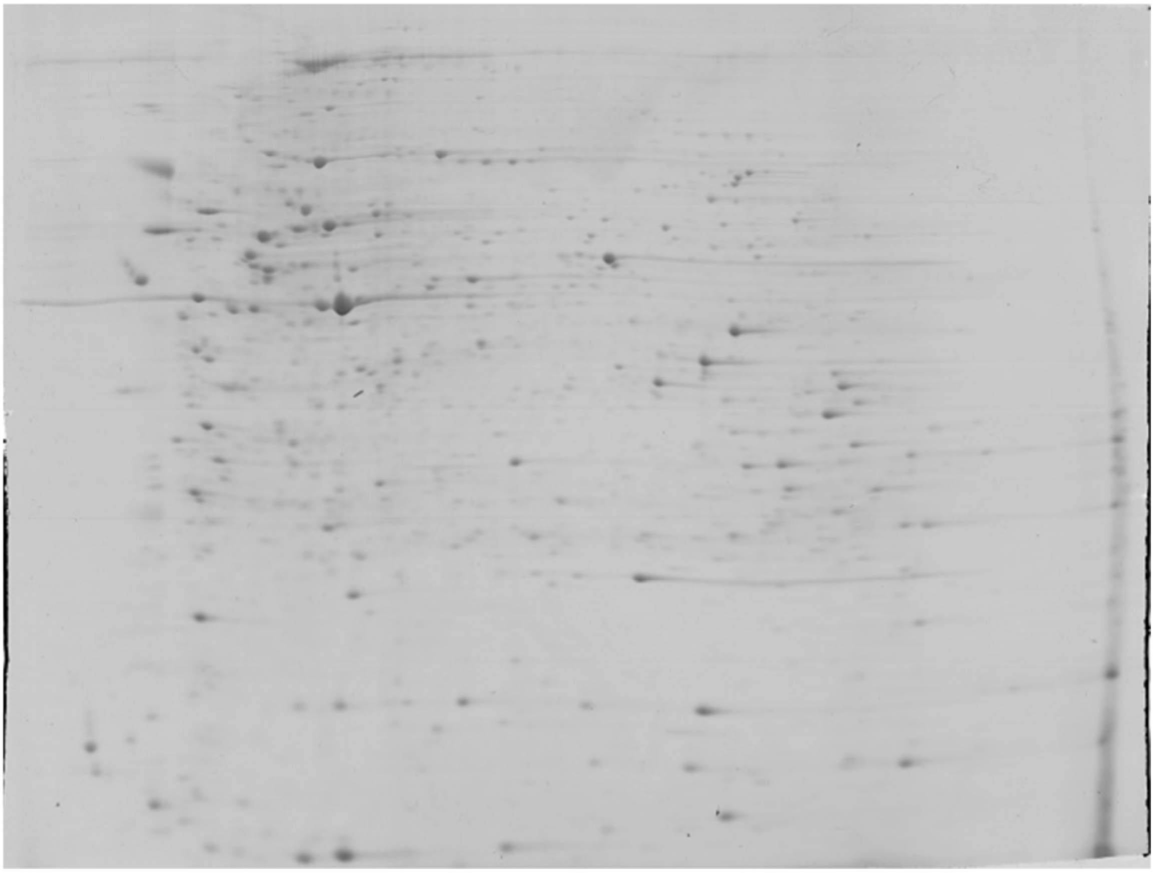

**C3D4 (**indicates culture number and culture day)

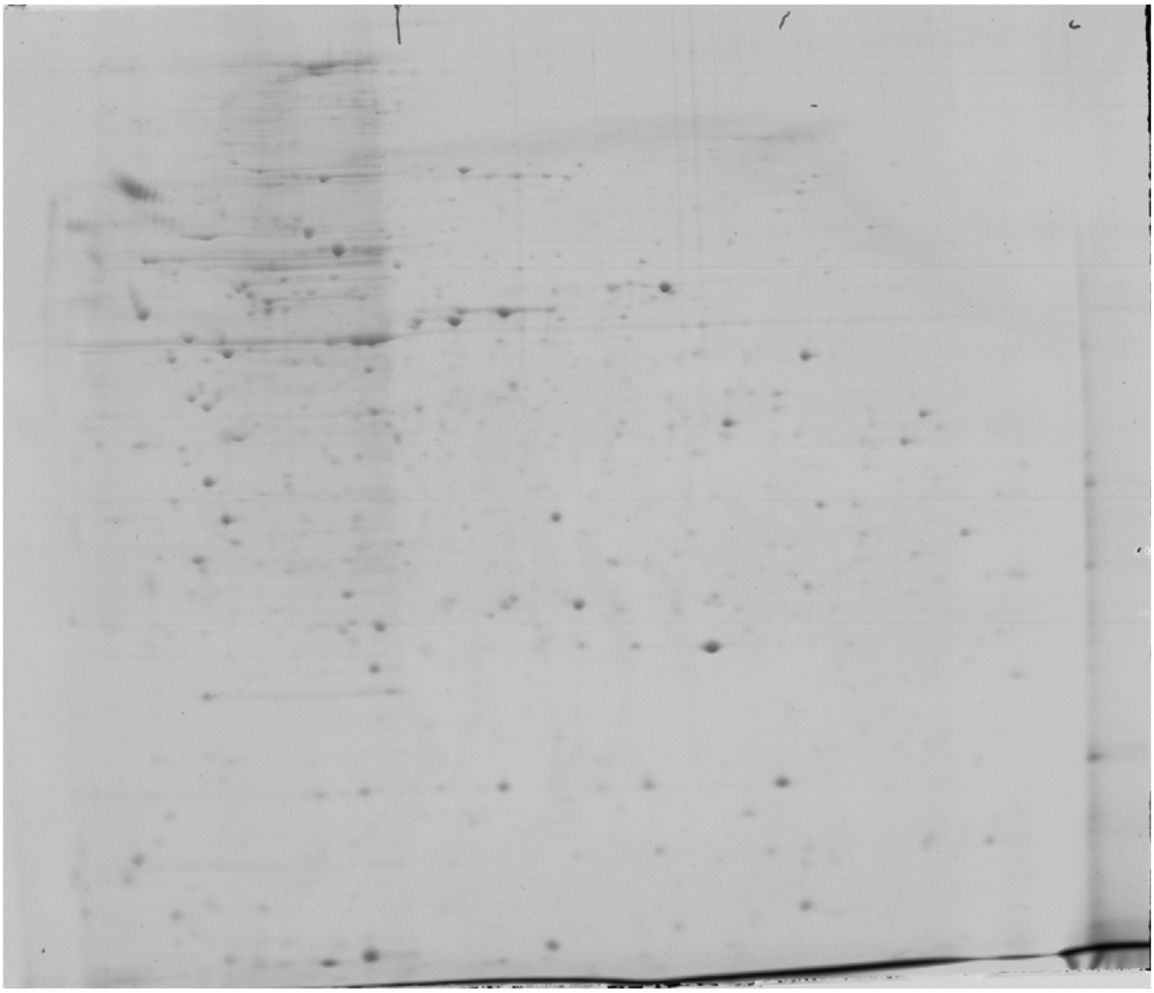

**C2D4**

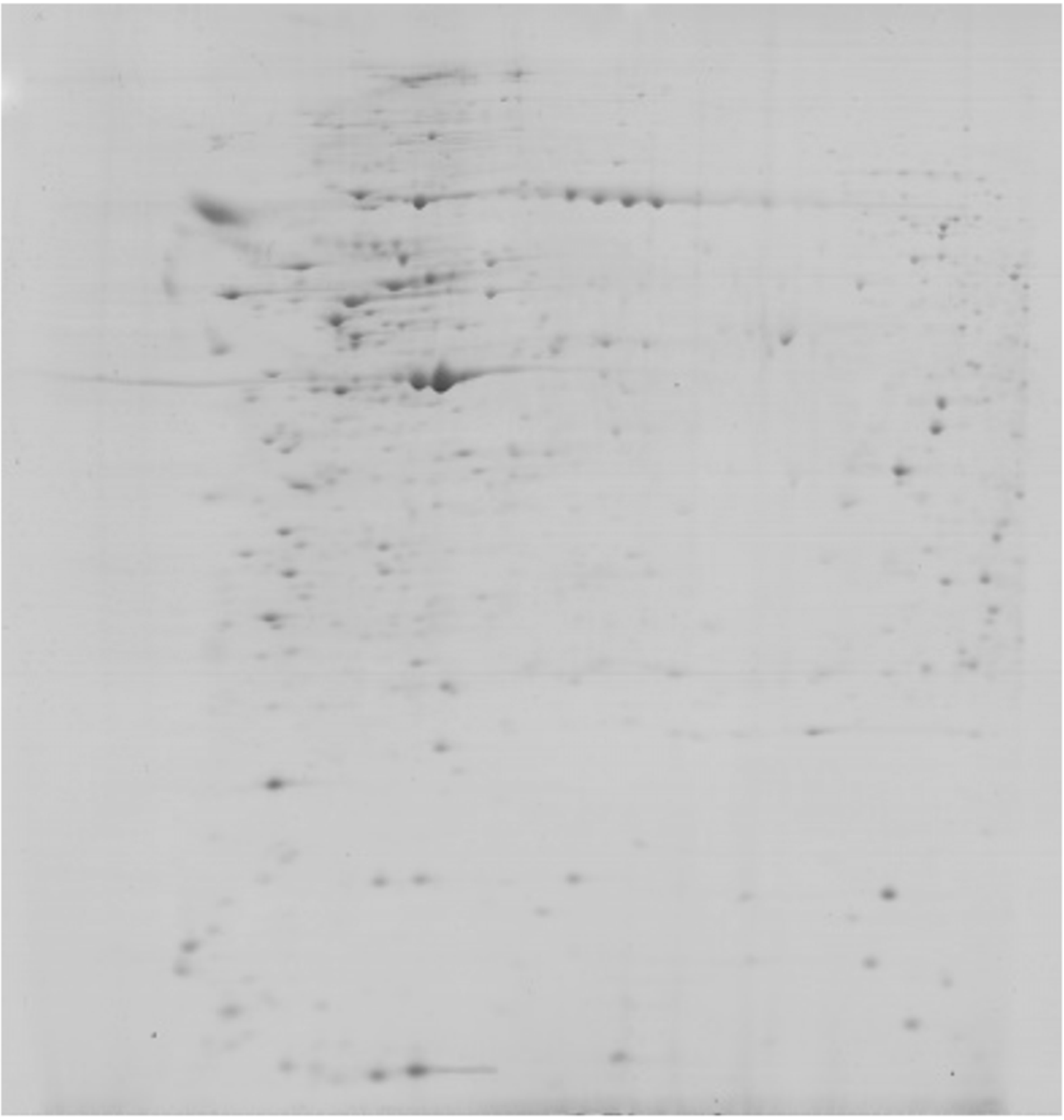

**C1D4**

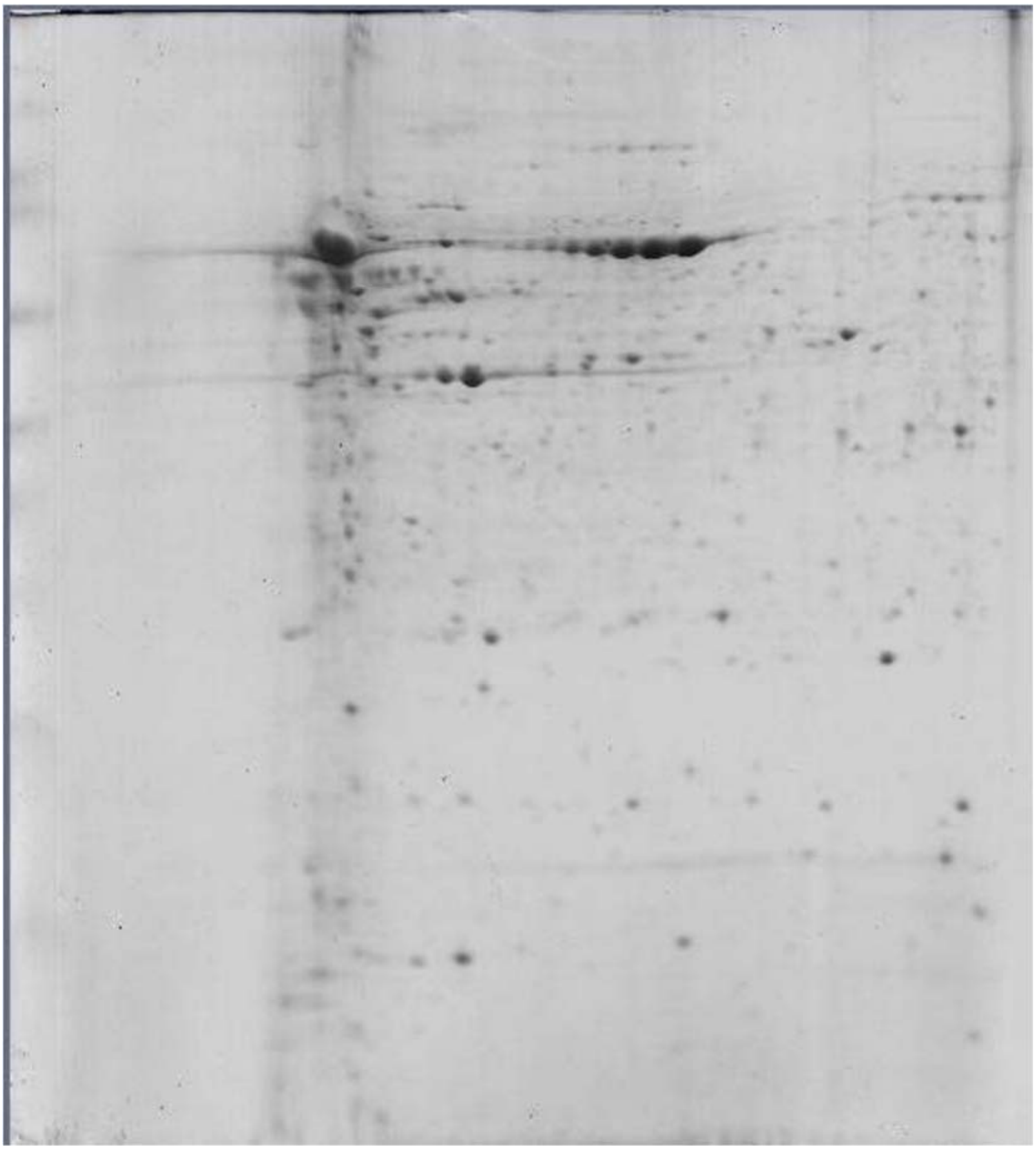

**C4D4**

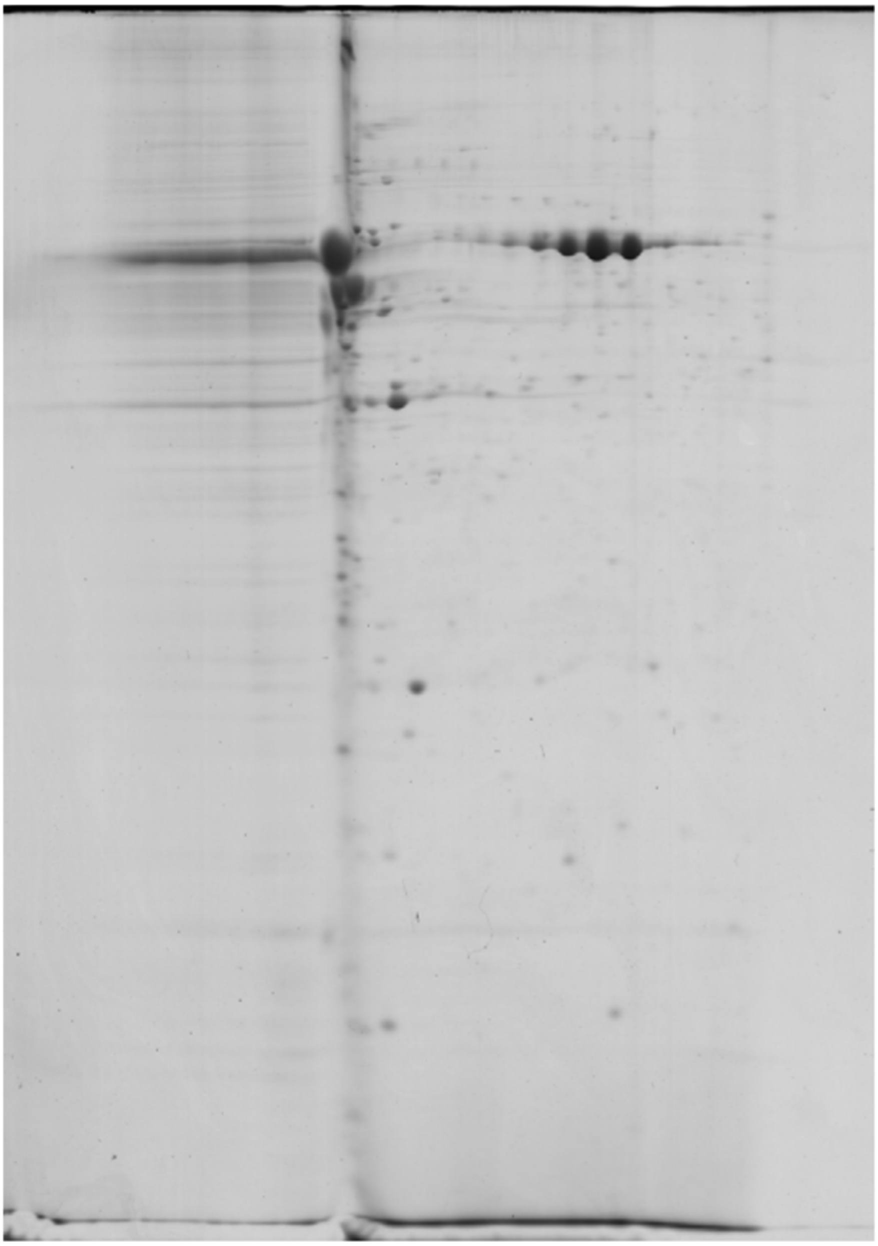

**C5D4**

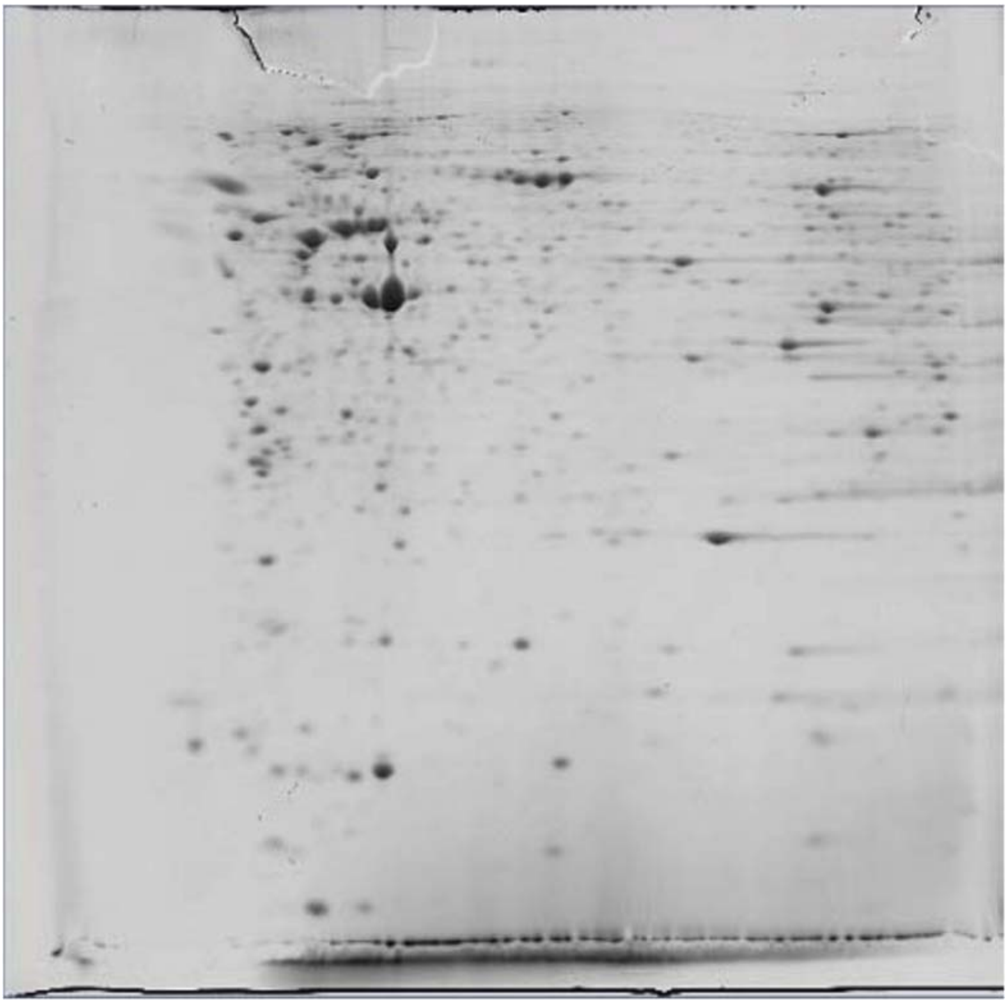

**C4D8**

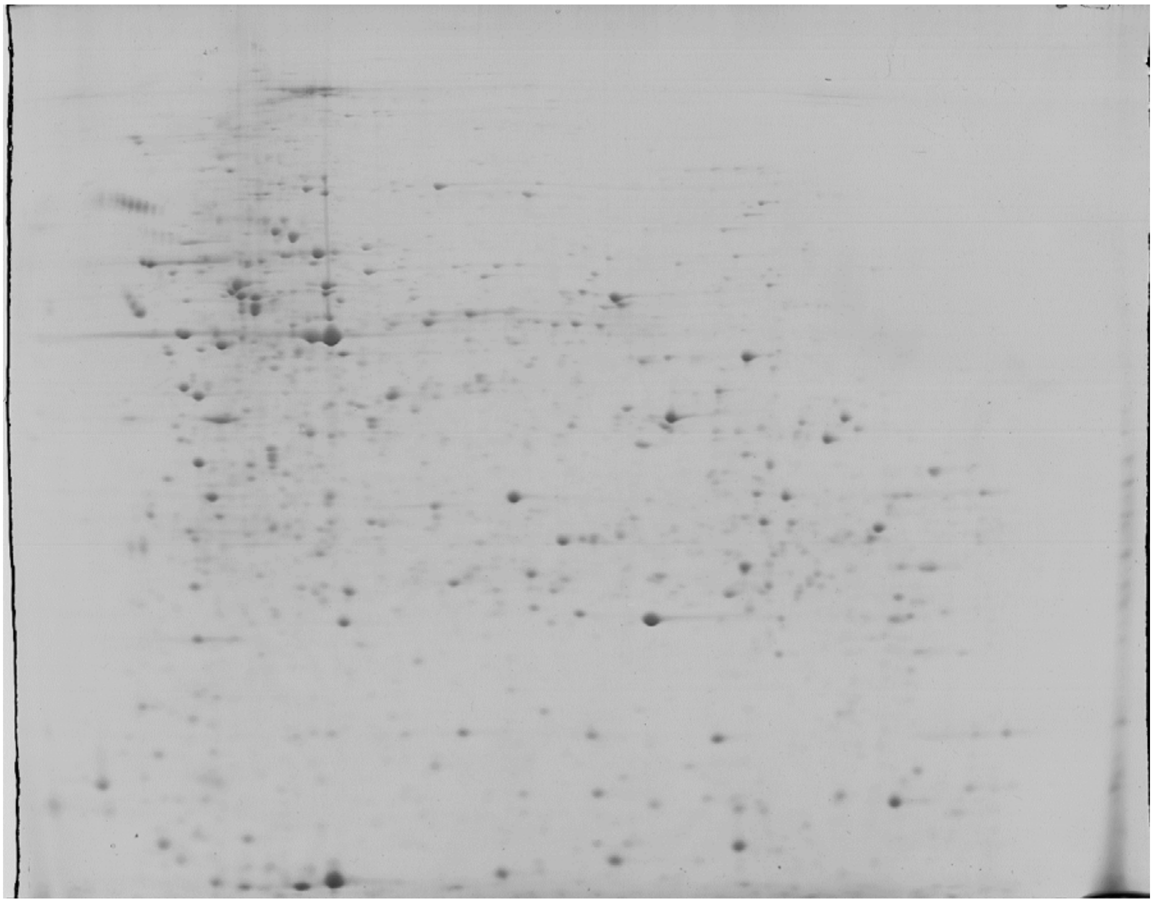

**C5D8**

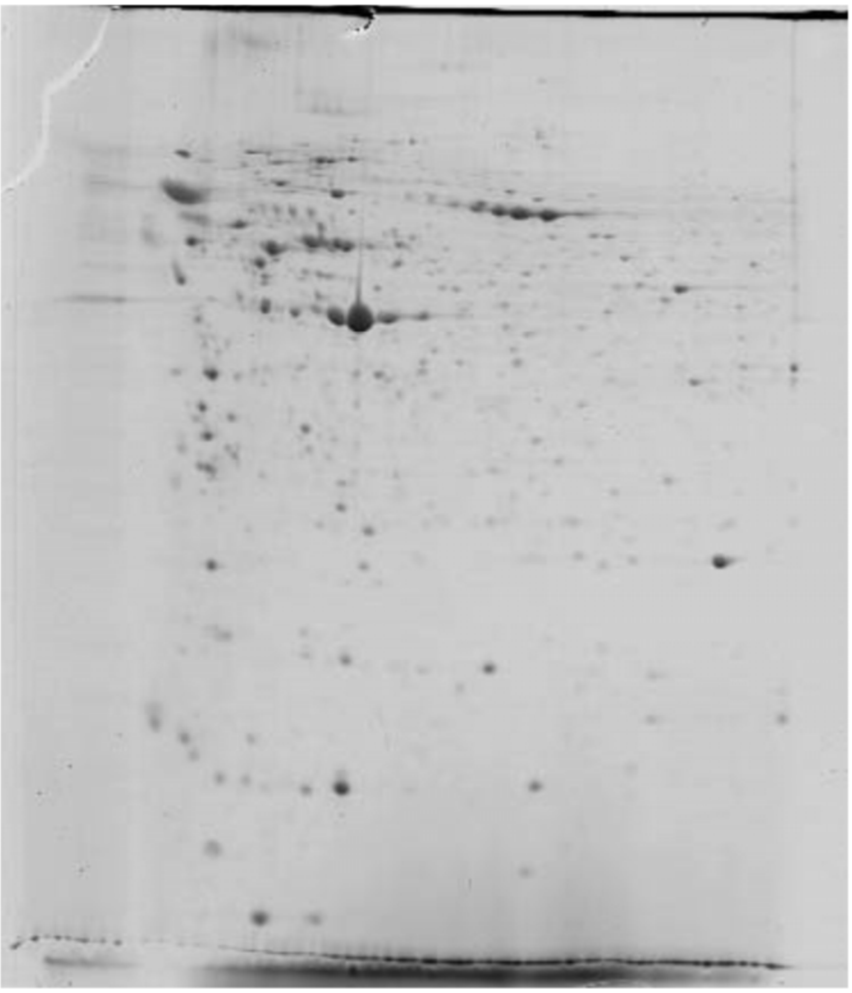

**C3D8**

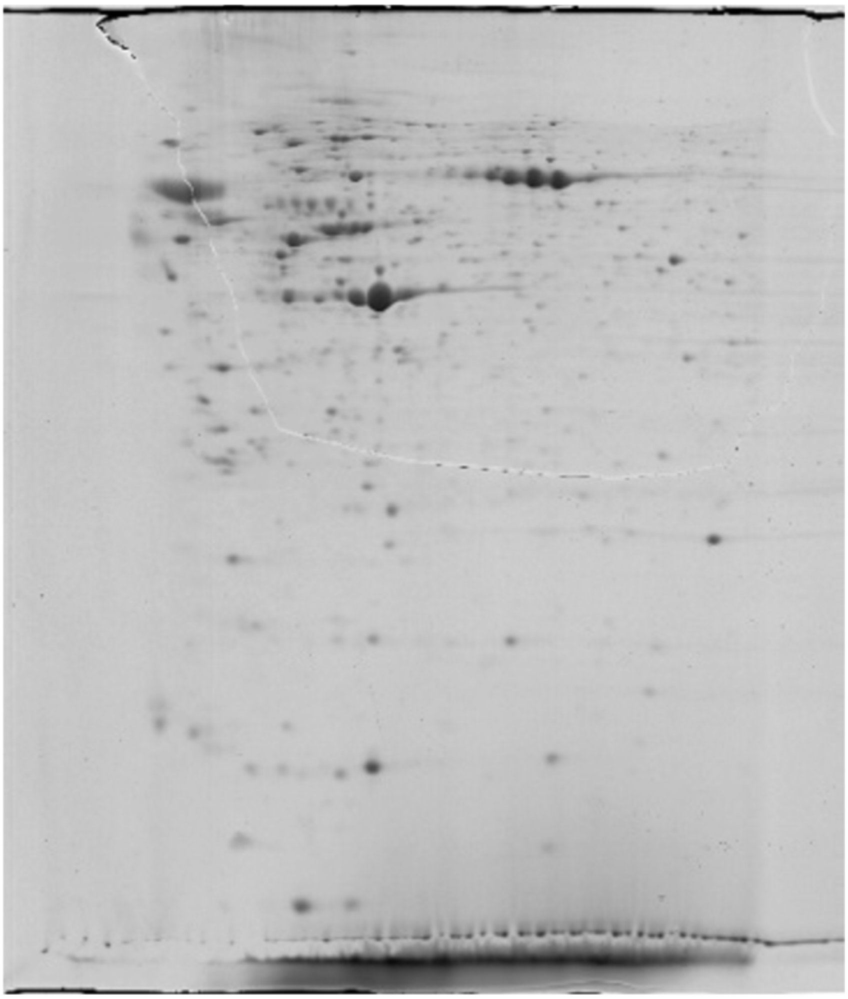

**C2D8**

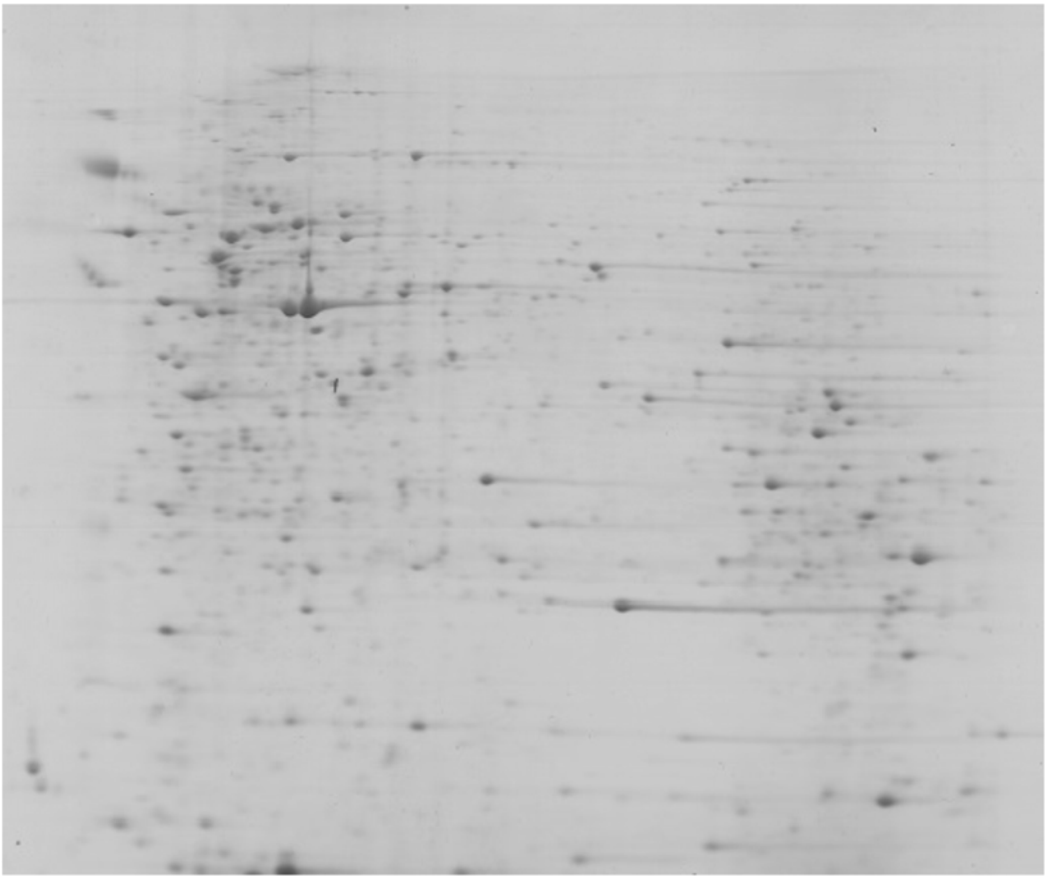

**C1D8**

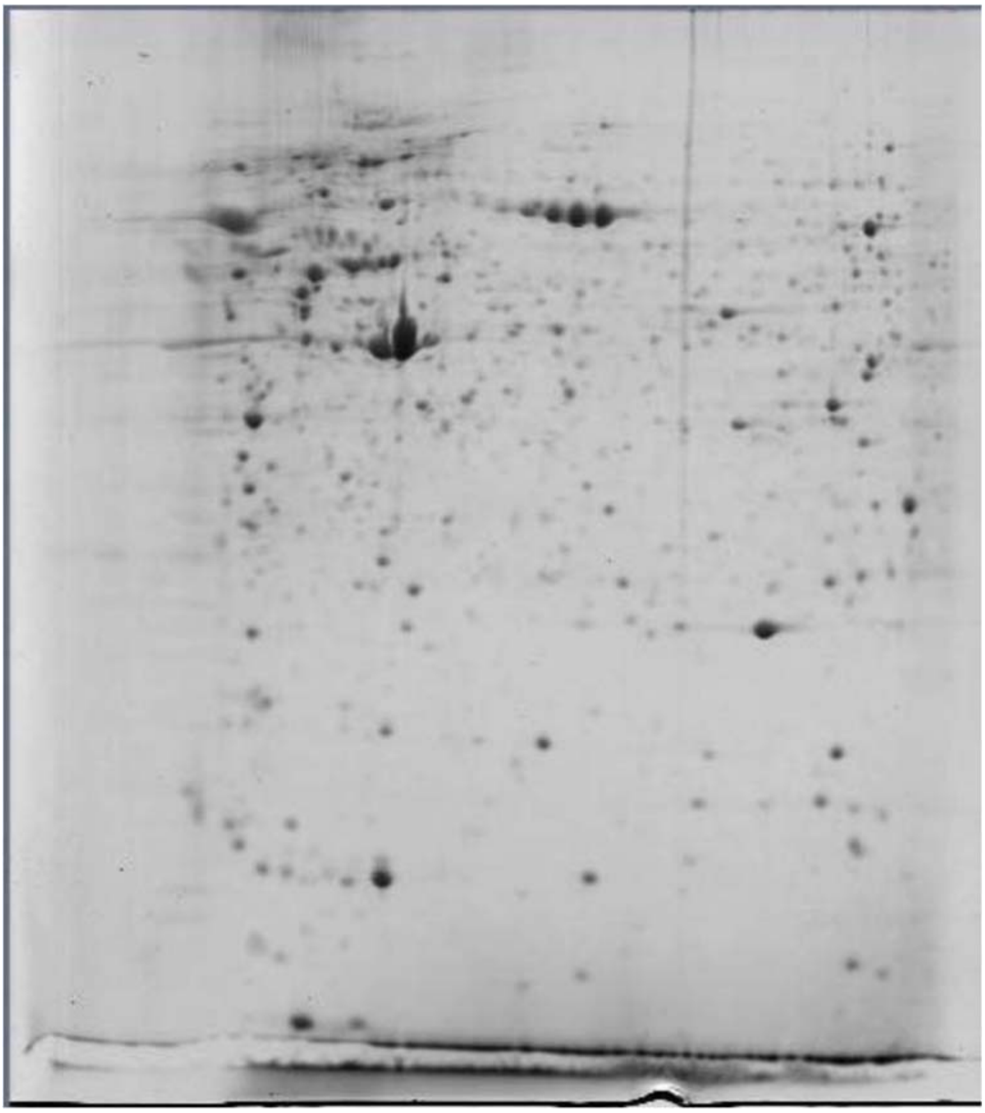

**C3D12**

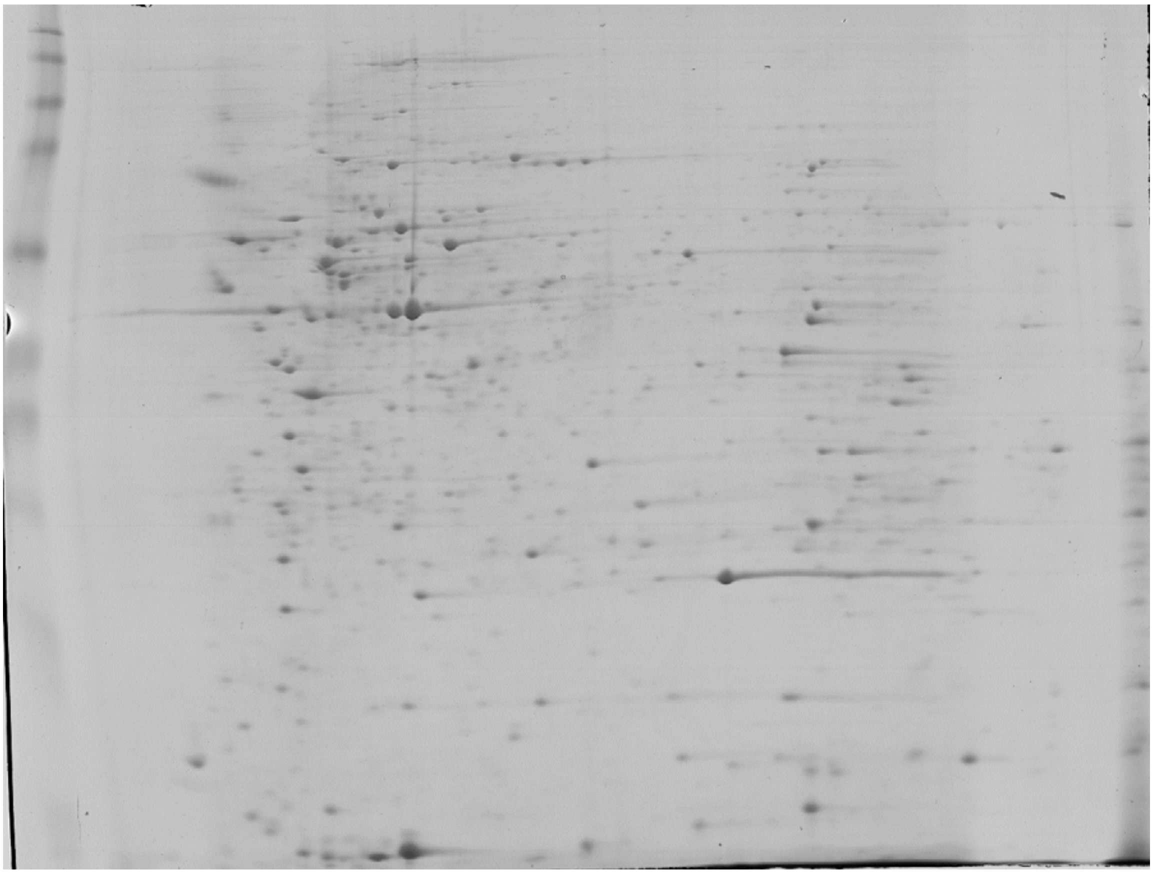

**C4D12**

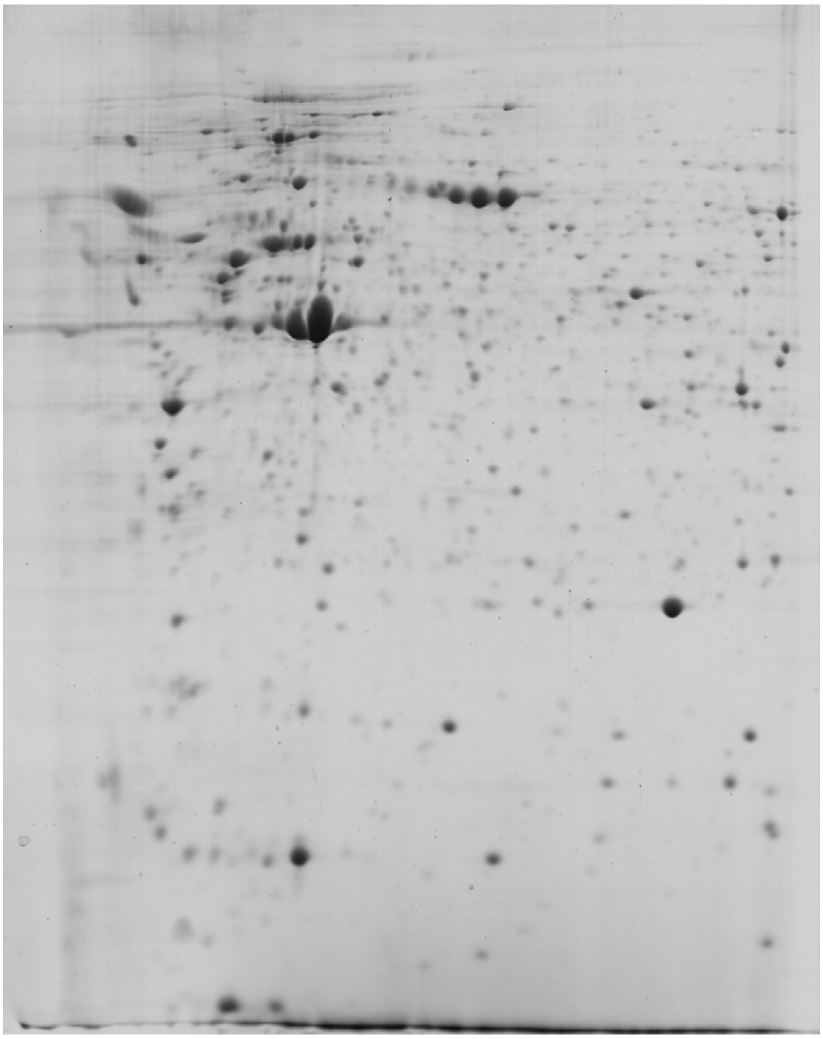

**C2D12**

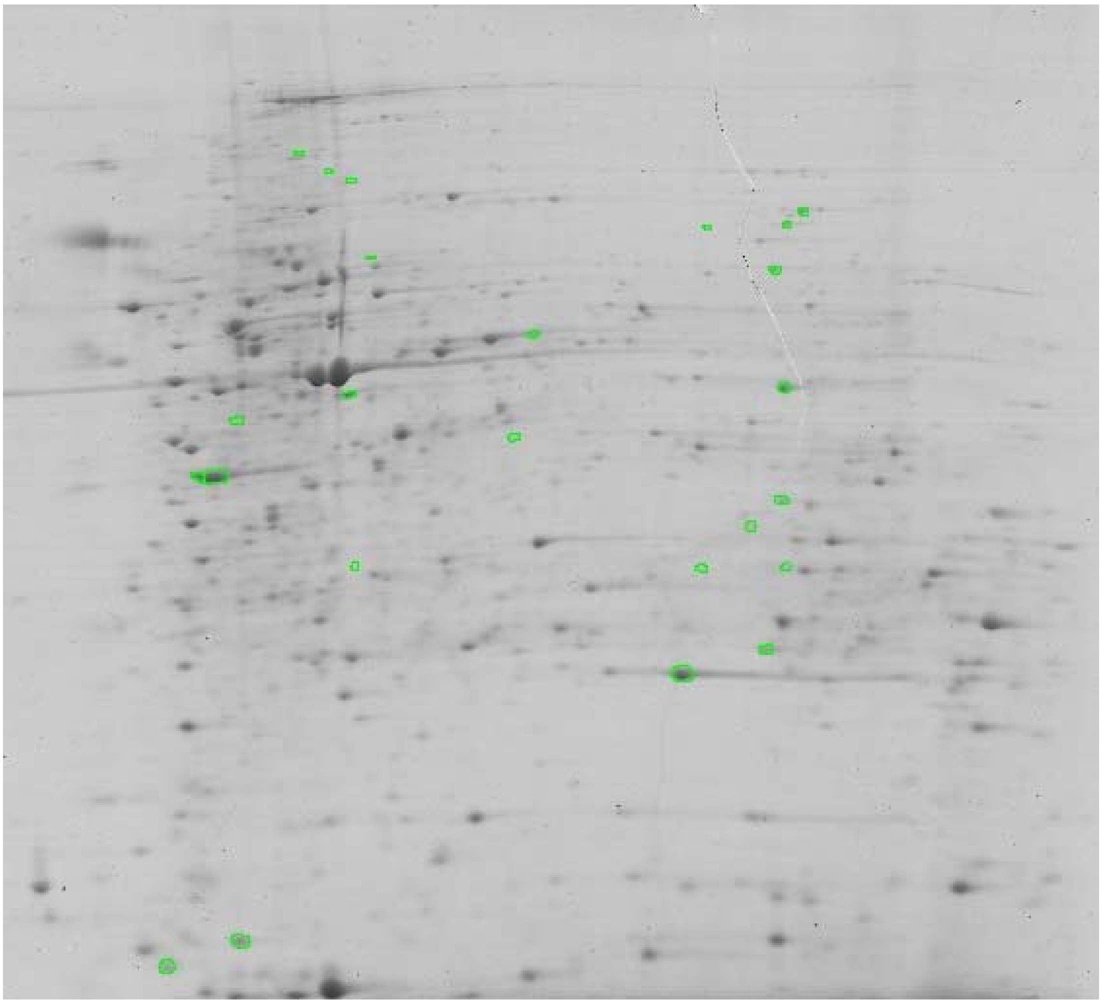

**C1D12**

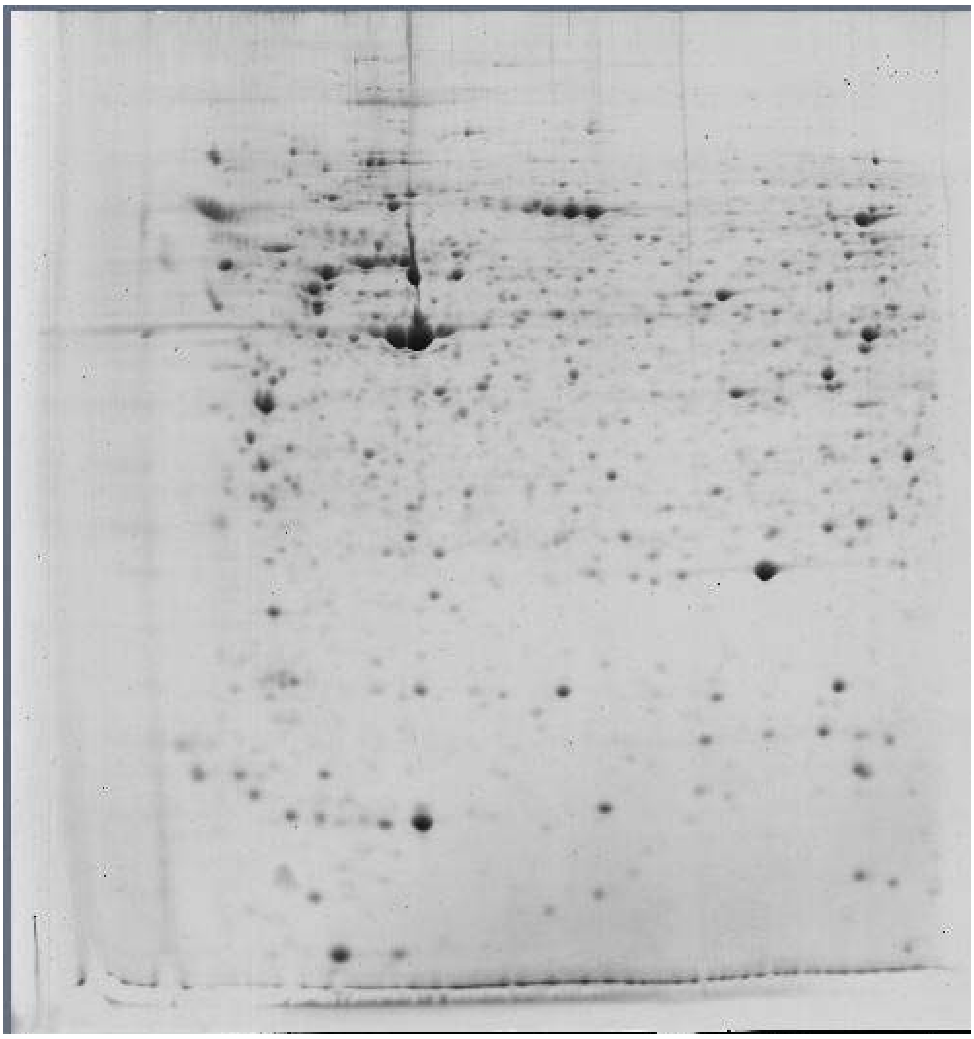

**C5D12**

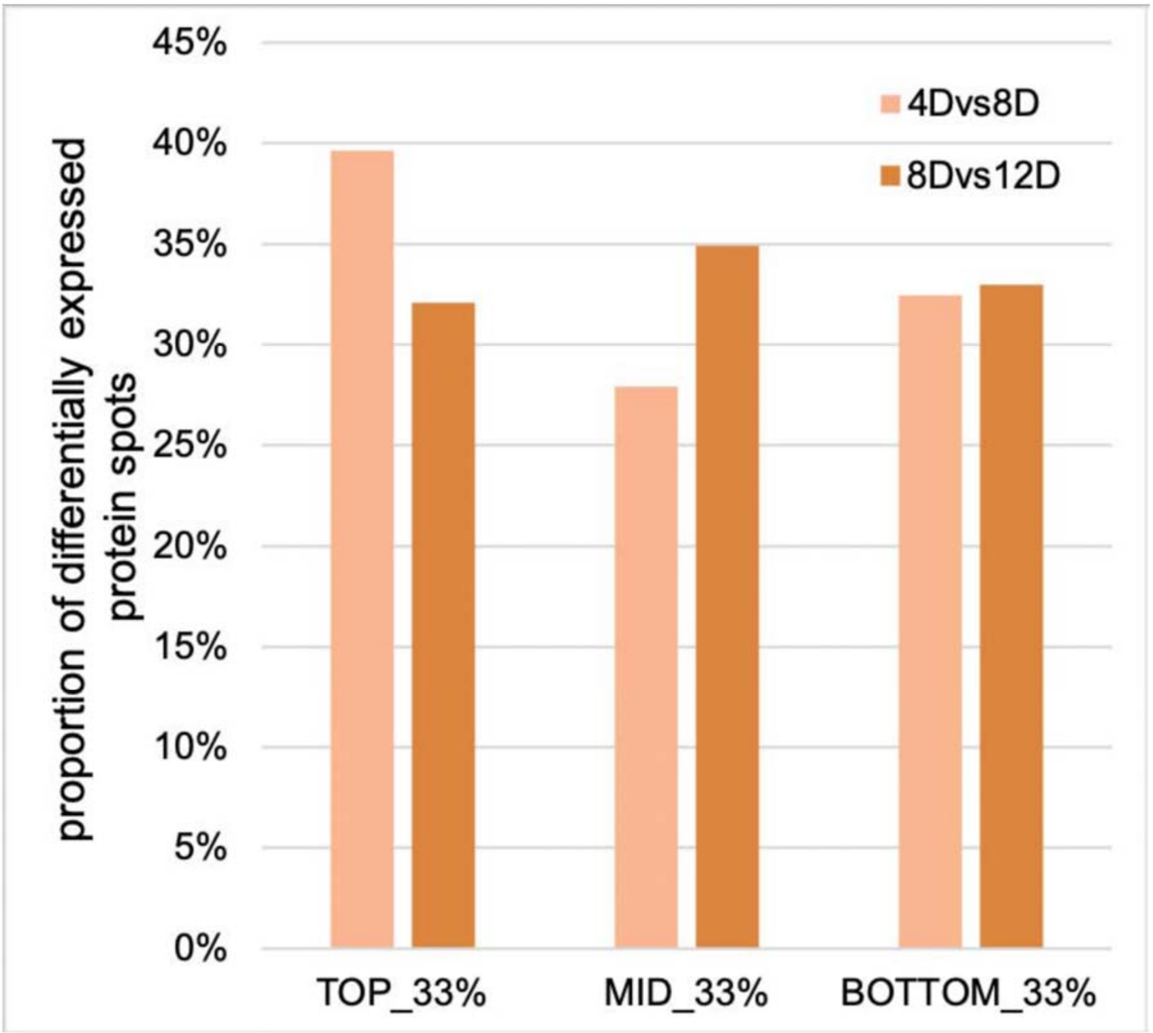


**Figure S2:** Distribution of differentially expressed proteins according to their mean relative abundance across all spots detected in gels at day 8 (8D).
